# Measuring Estuarine Total Exchange Flow From Discrete Observations

**DOI:** 10.1029/2022JC018960

**Published:** 2022-10-22

**Authors:** E. P. Lemagie, S. N. Giddings, P. MacCready, C. Seaton, X. Wu

**Affiliations:** ^1^ Pacific Marine Environmental Laboratory NOAA Seattle WA USA; ^2^ Scripps Institution of Oceanography UCSD La Jolla CA USA; ^3^ University of Washington Seattle WA USA; ^4^ Columbia River Inter‐Tribal Fish Commission Portland OR USA; ^5^ Shanghai Jiao Tong University Shanghai China

**Keywords:** total exchange flow, estuary dynamics, sampling strategies, mooring placement

## Abstract

The exchange between estuaries and the coastal ocean is a key dynamical driver impacting nutrient and phytoplankton concentrations and regulating estuarine residence time, hypoxia, and acidification. Estuarine exchange flows can be particularly challenging to monitor because many systems have strong vertical and lateral velocity shear and sharp gradients in water properties that vary over space and time, requiring high‐resolution measurements in order to accurately constrain the flux. The total exchange flow (TEF) method provides detailed information about the salinity structure of the exchange, but requires observations (or model resolution) that resolve the time and spatial co‐variability of salinity and currents. The goal of this analysis is to provide recommendations for measuring TEF with the most efficient spatial sampling resolution. Results from three realistic hydrodynamic models were investigated. These model domains included three estuary types: a bay (San Diego Bay), a salt‐wedge (Columbia River), and a fjord (Salish Sea). Model fields were sampled using three different mooring strategies, varying the number of mooring locations (lateral resolution) and sample depths (vertical resolution) with each method. The exchange volume transport was more sensitive than salinity to the sampling resolution. Most (>90%) of the exchange flow magnitude was captured by three to four moorings evenly distributed across the estuarine channel with a minimum threshold of 1–5 sample depths, which varied depending on the vertical stratification. These results can improve our ability to observe and monitor the exchange and transport of water masses efficiently with limited resources.

## Key Points

- A threshold of 1–5 sample depths was needed to measure the exchange flow
- Most (>90%) of the exchange flow magnitude was captured by 3–4 moorings spread across the channel
- The exchange volume transport was more sensitive than salinity to the sampling resolution

## 1 Introduction

The exchange between estuaries and the coastal ocean is a key dynamical driver impacting biogeochemical patterns such as nutrient and phytoplankton concentrations within the estuary (e.g., Boyer et al., 2002; Brown & Ozretich, 2009) and in the coastal ocean (e.g., Davis et al., 2014). This exchange can regulate estuarine residence time, hypoxia, and acidification (e.g., MacCready et al., 2021; O'Callaghan et al., 2007). Estuaries deliver terrigenous material to the ocean including sediment, larvae, and pollutants. Estuaries can also impact coastal circulation by delivering river runoff into the coastal margins (e.g., Banas et al., 2009; Giddings et al., 2014; Mazzini et al., 2014). Our ability to accurately observe the exchange at the estuary‐ocean interface is therefore important to understanding the physics, biology, chemistry, and coupling of estuarine and coastal ecosystems. Exchange flows are also important mechanisms in the transport and mixing of water masses through inland seas (e.g., Becherer et al., 2016; Burchard & Badewien, 2015) and through straits connecting marginal seas and the coastal ocean (e.g., Reissmann et al., 2009).

Estuarine exchange is typically thought of as being driven by buoyancy via longitudinal gradients in density (MacCready & Geyer, 2010), although other mechanisms are also important and can dominate over the buoyancy driven exchange flow such as tidal asymmetry (Burchard & Hetland, 2010). Other mechanisms can significantly complicate this simple picture and contribute to estuarine exchange flow including lateral circulation (e.g., Lerczak & Geyer, 2004), bathymetric complexity (e.g., Geyer et al., 2020), tidal mixing (e.g., Cheng et al., 2011; Griffin & LeBlond, 1990), the Earth's rotation (e.g., Valle‐Levinson et al., 2003), and more. Estuarine exchange is particularly challenging to monitor because many estuaries have strong vertical and lateral velocity shear and salinity gradients that vary over space and time, requiring high resolution measurement and strategic extrapolation in order to accurately constrain the flux.

Regardless of the estuary size, depth, transport, degree of stratification, and of the dominant forcing mechanisms, exchange flow is governed by the Knudsen relations which use mass and salt conservation to show that the exchange flow can be many times larger than the river flow (Burchard et al., 2018; Knudsen, 1900). The Knudsen (1900) theorem calculates the inflow (*Q*_in_) and outflow (*Q*_out_) and representative salinities (*S*_in,out_) assuming the exchange flows occur in layers of constant salinity. More recently the total exchange flow (TEF) method for computing the subtidal exchange parameters *Q*_in_, *Q*_out_, *S*_in_, *S*_out_ was proposed by MacCready (2011) and was updated to be more numerically accurate by MacCready et al. (2018a, 2018b) and Lorenz et al. (2019). TEF uses isohaline coordinates (Walin, 1977) to track the exchange flow, thus extending the Knudsen (1900) theorem to conditions with time‐variable stratification and flow, incorporating both subtidal and tidal fluxes (Chen et al., 2012). TEF provides detailed information about the salinity structure of the exchange flow, can identify multiple layers of exchange, can be applied in inverse estuarine conditions (Lorenz et al., 2019, 2020), and can be directly related to mixing (MacCready et al., 2018a, 2018b).

TEF has been widely applied in estuarine research and exchange flows more generally. The TEF framework has been used to determine freshwater fluxes from a small groundwater‐driven estuary (Ganju et al., 2012), to estimate estuarine residence times (Lemagie & Lerczak, 2014; Sutherland et al., 2011), to examine the relationship between exchange flow and mixing (Wang et al., 2017), and study seasonal variability (Conroy et al., 2020; Giddings & MacCready, 2017), among others. Most of the aforementioned examples are modeling studies, with the exception of Ganju et al. (2012). There are also analyses of salinity flux from observations that do not use the TEF framework (e.g., Lerczak et al., 2006; MacDonald & Horner‐Devine, 2008), but calculations of salt flux from observations are limited due to the large data requirement to resolve the temporal and spatial co‐variability in salinity and currents as well as a lack of knowledge regarding flux errors when undersampling occurs.

The goal here is to provide recommendations for applying TEF to *in situ* observations, specifically to understand the most efficient spatial sampling resolution and the percent of the exchange flow captured under various strategies. This paper examines TEF calculated from sub‐sampling realistic numerical models, representative of moorings in a channel, compared to TEF calculated from the full model resolution in order to compare how quickly the two estimates converge as the number of moorings increase. Three estuaries were studied in order to span much of the parameter space of estuarine characteristics. The objectives of this study were (a) to test how TEF converged for different sampling resolutions; (b) to examine how this varied between estuaries and sampling strategies; (c) to attempt to outline best practices for how many moorings and instruments would be required to quantify TEF from observations; and (d) to understand flux errors (magnitude and potential bias) when a cross‐section is under‐sampled. The three realistic estuary models, details of the TEF calculation, and the sampling methods are described in the methods Section 2. The current and salinity patterns characteristic of each estuary and individual cross‐section are included in Section 3. The rest of the results are organized into sections based on the various sampling approaches: evenly distributed moorings (Section 4), strategically distributed moorings (Section 5), and a case study designed to approximate a simple observational approach (Section 6).

## 2 Methods

### 2.1 Realistic Hydrodynamic Models

**Figure 1:**
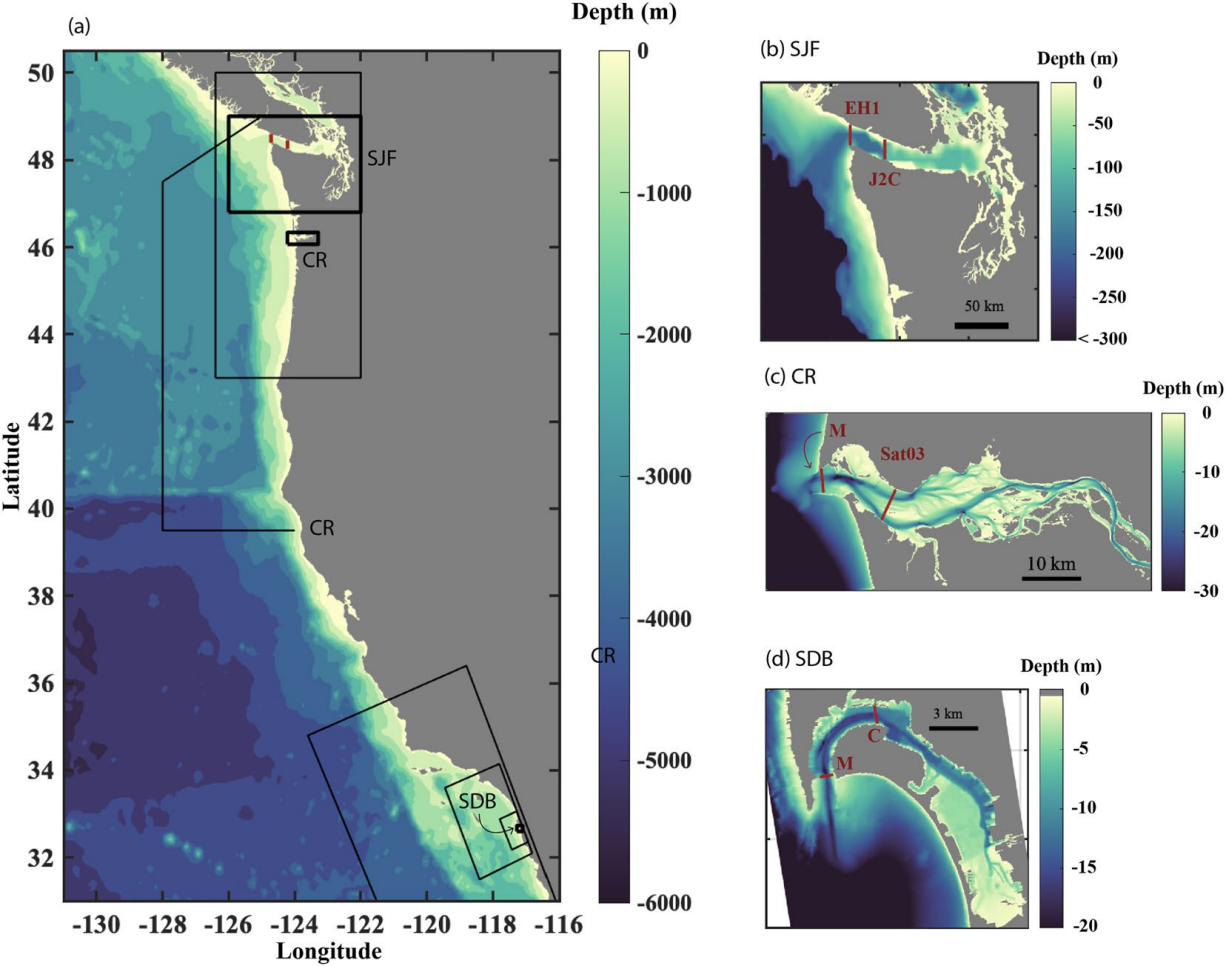
(a) The US west coast with the region around each realistic numerical model outlined with thin black boxes. Corresponding with the thick boxes in (a) more detail is shown around the estuarine cross‐sections in (b) the Strait of Juan de Fuca, SJF, (c) the Columbia River, CR, and (d) San Diego Bay, SDB. Red lines mark each of the cross sections examined as part of this study. Colors denote bathymetric depth. Note the lateral and color scales vary between maps.

Realistic hydrodynamic numerical models of three estuaries and their adjacent coastal regions were used (Figure 1). These span different estuary types and geometries and include a small bay (San Diego Bay), salt‐wedge (Columbia River), and large fjord (Strait of Juan de Fuca in the Salish Sea; e.g., Geyer & MacCready, 2014). Results were extracted hourly from two across‐channel sections in each model over a full year of simulation time in order to resolve the tides and capture seasonal variation. Extracted data is available on‐line (Lemagie et al., 2022). Further details about each model are outlined in the following paragraphs.

The Salish Sea model including the Strait of Juan de Fuca employed the Regional Ocean Modeling System (ROMS, Shchepetkin & McWilliams, 2005). Simulations from 2004 to 2007 were developed by the University of Washington Coastal Modeling Group (Giddings et al., 2014; MacCready et al., 2009; Sutherland et al., 2011). The model was forced with realistic river flow, tides, wind stress, surface heat flux, and open boundary conditions (e.g., Giddings et al., 2014) with initial and open boundary values for tracers, subtidal velocity, and subtidal surface height from the Navy Coastal Ocean Model (NCOM) (Barron et al., 2006; Kara et al., 2006). The domain spans the inland waters of the Salish Sea (including Puget Sound, the Strait of Georgia, and the Strait of Juan de Fuca) and coastal ocean from 43N to 50N and 200 km offshore with a horizontal resolution of 1.5 km at the coast to 4.5 km far offshore. There were 40 sigma layers with enhanced vertical resolution near the surface and bottom. This analysis focuses on data extracted from 2005 at two cross‐sections spanning the Strait of Juan de Fuca (SJF): near the ocean entrance (SJF_EH1_), and 16 km upstream (SJF_J2C_; Figure 1). These sections correspond with previous analyses and validation of the model results (Giddings et al., 2014). At SJF_EH1_ and SJF_J2C_ the channel is 22.1 and 21.5 km wide, respectively, and 73 and 60 m deep (Table 1). Model skill was high (≥0.92) relative to observed currents, tidal sea surface elevation, salinity and temperature, although overall slightly too salty and cold (by ∼1.5 psu and ∼0.5*°* *C*) within the Salish Sea (Giddings et al., 2014). Most pertinent to this study, the exchange flow through the Strait of Juan de Fuca compared well with observations and was insensitive to model resolution (Giddings & MacCready, 2017).

**Table 1:** Description of Each Model Simulation and Cross‐Section, Including the Latitude and Longitude, Simulation Year, Maximum Cross‐Section Width *W*, Number of Grid Points Across the Section *I* and Mean Grid Width Δ*x*, Maximum Cross‐Section Depth *H*, Number of Vertical Layers *J* and the Mean Depth of Each Layer Δ*z*

Run	lat,lon	Year	*W* (km)	*I*	Δ*x* (m)	*H* (m)	*J*	Δ*z* (m)
SJF^a^ (SJF_EH1_)	(−124.71,48.49)	2005	22.1	14	1,578 ± 24	258	40	4.1 ± 2.6
SJF (SJF_J2C_)	(−124.21,48.35)	2005	21.5	14	1,537 ± 12	209	40	3.8 ± 2.2
CR^b^ (CR_M_)	(−124.04,46.23)	2012	4.2	41	102	18	37	0.3 ± 0.1
CR (CR_Sat03_)	(−123.94,46.20)	2012	5.7	58	98 ± 0.5	16	37	0.2 ± 0.1
SDB^c^ (SDB_M_)	(−117.23,32.69)	2016	0.5	16	29 ± 1 m	19	10	1.1 ± 0.8
SDB (SDB_C_)	(−117.20,32.72)	2016	1.2	14	82 ± 0.7 m	14	10	1.1 ± 0.8

^a^ SJF = Strait of Jaun de Fuca.

^b^ CR = Columbia River.

^c^ SDB = San Diego Bay.

The Columbia River (CR) simulation used the unstructured grid, semi‐implicit finite‐element model (SELFE; Zhang & Baptista, 2008) version 4.0.1. Results for these analyses were accessed through the Columbia River Inter‐Tribal Fish Commission Coastal Margin Observation and Prediction Program (CRITFC‐CMOP; stccmop.org). Temperature, salinity, and water elevations were imposed at the oceanic boundary from the Navy Coastal Ocean Model (NCOM) for years 1999–2012 (analysis here focuses on the year 2012; Barron et al., 2006). The domain extended from 39N to 50N and ∼300 km in the offshore direction with horizontal resolution from tens of meters in the estuary and river to 3 km in the ocean (Karna & Baptista, 2016; Karna et al., 2015). The vertical grid consisted of 37 sigma levels between sea level and 100 m datum and an equipotential z‐grid below 100 m. Data were extracted from two cross‐sections: the river mouth (CR_M_) and 14 km upstream at site Saturn‐03 (CR_Sat03_), to match previous studies where model validation was performed (Figure 1; e.g., Karna et al., 2015). At CR_M_ and CR_Sat03_ the channel is 4.2 and 5.7 km wide and 18 and 16 m deep, respectively (Table 1). The model demonstrated high skill compared to long term observations, particularly outside of high discharge and neap tide conditions which are estimated to occur only 16% of the time (Karna & Baptista, 2016).

The San Diego Bay (SDB) and adjacent coastal dynamics were simulated using the Coupled Ocean‐Atmosphere‐Wave‐Sediment‐Transport (COAWST) model system (Kumar et al., 2012; Warner et al., 2010) to represent the surfzone and shelf circulation (Wu et al., 2020, 2021). This model grid sits within three one‐way nested parent models using ROMS (Shchepetkin & McWilliams, 2005) and is coupled with the Simulating Waves Nearshore (SWAN) model to include surface gravity waves (Booij et al., 1999). Boundary and initial conditions for the outermost domain were from the California State Estimate (CASE) solution (Marshall et al., 1997) with tides from the Advanced CIRculation tidal database (Westernik et al., 1993) and surface forcing from the North American Mesoscale Forecast (NAM) and the Coupled Ocean‐Atmosphere Mesoscale System (COAMPS). The largest grid extends between 29N and 36N and over 500 km offshore with 2 km horizontal resolution, which is downscaled to the finest grid with horizontal resolution from 8 m near the coast to 110 m at the western boundary, and has 10 stretched vertical sigma levels (Wu et al., 2020, 2021). This study focuses on 2016 results at the estuary mouth (SDB_M_) and 4 km upstream (SDB_C_; Figure 1). At SDB_M_ and SDB_C_, the channel is 0.5 and 1.2 km wide and up to 19 and 14 m deep, respectively (Table 1). This model has not been rigorously validated against observations of San Diego Bay, but exhibits circulation similar to prior observations (Largier et al., 1996).

### 2.2 Estuarine State Estimates

The vertical stratification index was used to characterize the water column at each cross section by

(1) $$\phi =-H^{-1}\int_{-H}^{0}\left(\rho -\bar{\rho }\right)gzdz,$$

where the overbar denotes a vertical mean. Vertical mean density $\bar{\rho }$ was computed

(2) $$\bar{\rho }=H^{-1}\int_{-H}^{0}\rho dz$$

following Simpson et al. (1981). *ϕ* gives an estimate of the potential energy of the water column relative to the mixed state such that in a vertically‐well mixed water column *ϕ* = 0. The influence of salinity and temperature on the density structure were computed by substituting $\rho =\rho \left(S\left(z\right),\bar{T}\right)$ and $\rho =\rho \left(\bar{S},T\left(z\right)\right)$, respectively. While available potential energy is another useful framework for understanding estuarine systems (e.g., MacCready & Giddings, 2016), *ϕ* is useful in this context because as *ϕ* approaches 0 *S*_in_ and *S*_out_ converge. *ϕ* was computed for each lateral column separately before calculating an area‐weighted cross‐sectional mean value.

The Kelvin number *K*_*e*_ and Ekman number *E*_*k*_ provide an estimate of the degree of horizontal and vertical variability in the currents (Valle‐Levinson, 2008) and may help predict how many moorings and vertical sample depths are needed to accurately constrain the exchange. The Kelvin number estimates the importance of Earth's rotation on the flow. Wide basins (*K*_*e*_ > 2) are more likely to have strong horizontal shear (Garvine, 1995). The Ekman number estimates the importance of vertical mixing (Kasai et al., 2000; Winant, 2004). Large Ekman number (*E*_*k*_ > 1) basins are likely to have strong horizontal shear regardless of their width (Valle‐Levinson, 2008). The Kelvin and Ekman numbers were calculated from the full resolution of model fields and then time‐averaged. $K_{e}=Wf{\left(g^{\prime }H\right)}^{-\frac{1}{2}}$ for estuary width *W*, depth *H*, reduced gravity *g*′, and coriolis parameter *f*. $E_{k}=A_{z}{\left(fH^{2}\right)}^{-1}$, where *A*_*z*_ is the flow's eddy viscosity. The eddy viscosity was not available from the Columbia River SELFE model, and *E*_*k*_ could not be explicitly calculated.

### 2.3 TEF Calculations

Subtidal exchange flow is calculated using isohaline coordinates following the TEF dividing salinity method (Lorenz et al., 2019). Following the TEF framework (MacCready, 2011), the net transport of a tracer *c* through a cross‐sectional area *A*(*S* > *S*′) determined by salinity *S*′ is defined as:

(3) $$Q^{c}\left(S,t\right)=\langle \int_{A\left(S>S^{\prime }\right)}cudA\rangle .$$

where *u* is the velocity normal to the cross‐section (positive values are into the estuary), *t* is time, and 〈〉 denotes a subtidal filter (here the Godin low pass filter, Thomson & Emery, 2014). A profile of the tracer exchange can also be determined by differentiation:

(4) $$q^{c}\left(S,t\right)=-\frac{\partial Q^{c}\left(S\right)}{\partial S}.$$

The transport profile was separated into distinct inflow and outflow layers (l) by finding the extrema in the *Q* ^*c*^ profiles, ignoring extrema below a certain noise threshold *Q*_thresh_ (Lorenz et al., 2019). *Q*_thresh_ was defined here as a fixed percentage of the maximum transport magnitude, *Q*_thresh_ = *Q*_percent_ ∗ max(|*Q* ^*c*^(*S*)|), where *Q*_percent_ = 0.01. The salinity values associated with the *Q* ^*c*^ extrema—along with the salinity endpoints *S*_min_, *S*_max_—made up the dividing salinities *S*_div_ and the transport in each layer was

(5) $$\Delta Q_{l}^{c}\left(t\right)=\int_{S_{\mathrm{div,l}}}^{S_{\mathrm{div,l}+1}}q^{c}dS.$$

Inflow was positive and layers were defined by the sign of the net transport:

(6) $$\begin{array}{c} \Delta Q_{\mathrm{in},a}^{c}\left(t\right)\equiv \Delta Q_{l}^{c}\left(t\right)>0, \end{array}\;\begin{array}{c} \Delta Q_{\text{out,}b}^{c}\left(t\right)\equiv \Delta Q_{l}^{c}\left(t\right)<0. \end{array}$$

Subscripts *a* and *b* are used to enumerate inflow and outflow layers respectively, following Lorenz et al. (2019). The net exchanges were defined by:

(7) $$\begin{array}{c} Q_{\mathrm{in}}^{c}\left(t\right)\equiv \sum_{a}\Delta Q_{\mathrm{in},a}^{c}\left(t\right), \end{array}\;\begin{array}{c} Q_{\mathrm{out}}^{c}\left(t\right)\equiv \sum_{b}\Delta Q_{\text{out,}b}^{c}\left(t\right). \end{array}$$

This summation transforms the results from *l* = 1: *L* individual layers into two layers, which does not greatly impact the result if the flow is predominantly two‐layered. The mean inflow and outflow salinities can be calculated by:

(8) $$\begin{array}{c} S_{\mathrm{in}}\left(t\right)=\frac{Q_{\mathrm{in}}^{S}\left(t\right)}{Q_{\mathrm{in}}\left(t\right)}, \end{array}\;\begin{array}{c} S_{\mathrm{out}}\left(t\right)=\frac{Q_{\mathrm{out}}^{S}\left(t\right)}{Q_{\mathrm{out}}\left(t\right)}. \end{array}$$

where the tracer, *c*, is the salinity, *S* and no superscript implies the volume flux only, $Q\left(S,t\right)=\langle \int_{A\left(S>S^{\prime }\right)}udA\rangle$. The above exchange flows and corresponding salinities are referred to as the TEF bulk values (e.g., Lorenz et al., 2019).

Currents and salinities were extracted hourly from each cross‐section. Since the SELFE model uses an unstructured grid, CR output were interpolated onto horizontally fixed straight cross‐sections at CR_Sat03_ and CR_M_, roughly matching the spatial resolution of the model grid. ROMS variables were extracted at the grid resolution. Velocities were rotated onto along‐ and across‐channel coordinates, defined by the angle of the cross‐section. The principle axis of the area‐averaged currents over the year were closely aligned with each cross‐section (e.g., Table 2). In order to avoid tidal aliasing, the start and end times were estimated by the timing of the spring tidal sea level maximum closest to each calendar end point.

**Table 2:** Oceanographic Characteristics, Including the Time and Area‐Averaged Mean Salinity, and Along‐Channel Current Magnitude, the Principle Axis of the Currents, and the Kelvin and Ekman Numbers Calculated at Each Cross Section From Unfiltered Time Series

Run	mean *S* psu	range *S* psu	mean |*u* (m s^−1^)	Princ.Ax. degrees	Ke	Ek
SJF (SJF_EH1_)	32.7	[20.4, 34.0]	0.34 ± 0.26	3	1.68	6.03 × 10^−4^
SJF (SJF_J2C_)	32.6	[25.5, 33.9]	0.36 ± 0.26	−22	1.81	1.07 × 10^−3^
CR (CR_M_)	21.8	[0.0, 33.1]	0.73 ± 0.54	26	0.46	
CR (CR_Sat03_)	8.7	[0.0, 32.3]	0.58 ± 0.71	4	0.85	
SDB (SDB_M_)	33.6	[32.2, 34.2]	0.26 ± 0.18	1	0.57	0.17
SDB (SDB_C_)	33.7	[32.9, 34.4]	0.16 ± 0.11	−14	1.36	0.22

*Note*. Along‐channel flow was defined as positive flowing into the estuary normal to the cross‐section and the principle axis is reported here as degrees counter‐clockwise from section normal (with the normal vector directed into the estuary).

### 2.4 TEF Sampling Strategies

**Figure 2:**
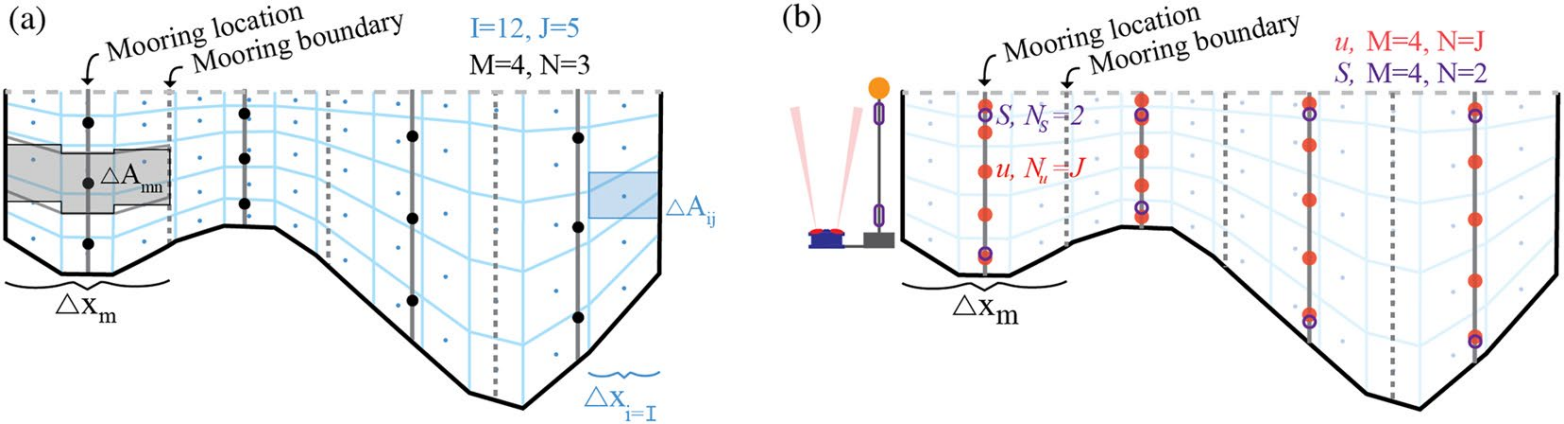
Illustration of the grid points used for three TEF calculations. In (a) thin blue lines indicate the full model resolution and blue dots mark cell centers where model fields were extracted, TEF_*IJ*_ = TEF_12,5_. Blue shading is a sample model grid cell area, which varies over time with sea level. Dark gray lines indicate example mooring locations for *M* = 4 with black dots indicating example mooring vertical sampling for *N* = 3. Dark gray dashed lines indicate the boundaries centered between simulated mooring locations for TEF_*MN*_ = TEF_4,3_. Gray shading demonstrates the mooring sample area for TEF_*MN*_ assuming that model fields *u* and *S* are constant across *σ*‐levels over distance Δ*x*_*m*_. In (b) the filled red dots and open purple circles indicate sample locations for currents and salinity, respectively for TEF_case_. TEF_*μJ*_ is not shown as it requires sequential mooring addition, however it is always sampled on the *I*, *J* grid (blue dots) but with *μ* ≤ *I* mooring locations potentially unevenly spaced.

Four methods of sampling the cross‐sectional fields and calculating TEF were compared: (a) using horizontal and vertical resolution from the *I*x*J* model grid to calculate TEF_*IJ*_; (b) using an *M* × *N* array of evenly spaced samples to calculate TEF_*MN*_ from an increasing integer number of “moorings” *M* evenly distributed across the channel width with *N* sample depths each, which were evenly distributed across the time‐averaged channel depth at each location *x* = *x*_*m*_ (e.g., Figure 2a); (c) using *μ* moorings with strategic placement of each mooring *μ* = 1, …, *I* determined by maximizing the correlation between TEF_*μJ*_ and TEF_*IJ*_; and (d) a case study TEF_case_ designed to imitate observations with a single bottom‐mounted Acoustic Doppler Current Profiler (ADCP) and salinity measurements near the surface and bottom of the water column at *M* evenly distributed mooring locations (e.g., Figure 2b). In cases 2–4, the width of the channel represented by each mooring is defined by the channel boundaries and the mid‐point between adjacent moorings (Figure 2). In cases 2 and 4, mooring locations shift as M varies so that all moorings are evenly spaced. Each of these methods is described in more detail in the following paragraphs.

One goal of this analysis was to identify the minimum number of moorings and samples required for various sampling approaches to converge to TEF_*IJ*_. An appropriate definition of convergence between these methods could depend on the specific application or research question. One comparison that demonstrated utility was to identify the threshold for which the magnitude of each TEF parameter consistently remained within ≤10% of the TEF_*IJ*_ value. However, since the observed salinity range was small relative to the magnitude of salinity values (Table 2), this 10% threshold for convergence was applied to salinity values normalized using the freshwater fraction. *S*_in,out_ were converted to equivalent freshwater fractions using the tidal maximum salinity over time at each cross‐section, *S*_max_ following ${FWF}_{\mathrm{in},\mathrm{out}}=\left(S_{\max}-S_{\mathrm{in},\mathrm{out}}\right){\left(S_{\max}\right)}^{-1}$.

#### 2.4.1 Full TEF, TEF_*IJ*_

TEF_*IJ*_ used the full vertical and horizontal resolution from the model grid. TEF_*IJ*_ represented the expected value which other estimates of TEF were hypothesized to converge toward at high sampling resolutions. Velocity and salinity fields were sampled at points (*x*_*i*_, *z*_*ij*_(*t*)) and the area represented by each sample was computed by Δ*A*_*i*,*j*_(*t*) = Δ*x*_*i*_Δ*z*_*ij*_(*t*). Subscripts *i* and *j* indicate the indices on the model grid in the across‐channel and vertical direction, respectively (e.g., Figure 2).

#### 2.4.2 Evenly Distributed Subsamples, TEF_*MN*_

TEF_*MN*_ used an *M*x*N* array of samples evenly distributed across the channel and throughout the water column (e.g., Figure 2a). This method was chosen to test the convergence of TEF_*MN*_ toward TEF_*IJ*_ as the number of moorings (*M* ≤ *I*) or sample depths (*N* ≤ *J*) were increased. This method is simple enough to be consistently applied in every case. However, when there is sharp bathymetric variability within width Δ*x*_*m*_, it is not obvious how to estimate area Δ*A*_*m*,*n*_(*t*). For example, on a steep slope a gridded area could either over‐estimate or under‐estimate the flux. To address this, two approaches were compared: the first method assumed that *u*, *S* were constant with depth over distance Δ*x*_*m*_ and the second assumed the profiles of *u*, *S* had a consistent shape over distance Δ*x*_*m*_ and were thus constant across *σ*‐levels as in Lerczak et al. (2006). The difference in the results between approaches was negligible. The results reported herein assume that *u* and *S* were constant along *σ*‐levels to estimate Δ*A*_*MN*_ (Equation 3) as illustrated on Figure 2a.

#### 2.4.3 Strategically Located Subsamples, TEF_*μJ*_

TEF_*μJ*_ was calculated by incrementally adding moorings in order based on identifying the mooring which contributed the largest improvement in the correlation coefficient between TEF_*μJ*_ and TEF_*IJ*_, similar to the approach of using maximum explained variance used by Wei et al. (2020). Lateral mooring placement was sampled at *μ* ≤ *I* grid locations while *J* sampling depths were included at each mooring location. Since TEF is a derived flux quantity, it was necessary to estimate the cross‐sectional area represented by each sample of *u* and *S*. This computation used linear interpolation assuming *u* and *S* were constant across *σ*‐levels, analogous to TEF_*MN*_. The lateral edges of the regions represented by each mooring *μ* were defined by the channel edges and the mid‐point between each mooring pair in the across‐channel direction. Importantly, the moorings are not necessarily evenly spaced in this approach. The sample area Δ*A*_*μJ*_ was calculated as the total model grid area at a given *σ*‐level between the lateral edges bounding each mooring *μ*.

#### 2.4.4 Case Study, TEF_case_

A case study was designed to imitate a sampling plan where *S* and *u* observations are not co‐located and are constrained by common instrument and deployment logistics (Figure 2b). *M* moorings were evenly distributed laterally over the cross‐section. At each mooring *u* was sampled at the full model resolution, to approximate having a bottom‐mounted ADCP and salinity was sampled 1 m off of the bottom and 1 m below sea level, to approximate having a bottom‐mounted sensor as well as one mounted from a surface float. From this sampling distribution two variations of vertical salinity interpolation were compared. In case A, *S* was linearly interpolated to the velocity sample depths (i.e., the model grid). In case B, a two‐layer system was assumed having well‐mixed surface and bottom layers each with constant *S*. The depth of the boundary between the well‐mixed layers was approximated by the mean depth of the 0‐crossing between inflow and outflow in the deepest part of the channel (50, 8, and 5 m at sections SJF_EH1_, CR_M_, and SDB_M_, respectively). With observations this interpolation could be calculated during the analysis stage using observed currents, therefore this estimate of the mixed layer depth does not rely on a priori knowledge. In reality, vertical patterns of currents and salinities may be decoupled, or may vary over time, however this simplified approach is applied for the case study to be most relevant to an observational study with limited to no a priori knowledge about the system. While not always dynamically appropriate, it is a reasonable simplified approach for many estuarine systems (e.g., Aristizábal & Chant, 2015; Lerczak et al., 2006). Tests with extrapolated currents in the top and bottom 10% of the water column, and 2–5 m from the bottom—to simulate ADCP limitations—had negligible impact on the results. Additional case studies mimicking other sampling strategies were not included since TEF_*MN*_ and TEF_case_ already span the parameter space for most sampling approaches and some approaches, such as shipboard transects, are typically limited in duration.

#### 2.4.5 Discrete Calculations

For the discrete calculation of Equation 3 applied to each TEF method the spatial coordinates were first converted to isohaline coordinates. The salinity range was defined by the minimum and maximum salinity sampled at each cross‐section over the year. This range was divided into *N*_bins_ = 500 evenly spaced salinity bins. The currents *u*, salinity *S*, and area *A* were interpolated onto the spatial grid defined for each method (e.g., Figure 2) and then mapped into these discrete salinity bins at each time prior to the calculation of TEF (Equation 3).

## 3 Exchange Flow

The estuaries and individual cross‐sections chosen for this study differ in the degree of stratification and shear, the range of seasonal and tidal variability, as well as in the channel width and bathymetric complexity. These features contribute to differences in TEF. Before presenting a comparison of the TEF calculated by different methods and resolutions, the characteristics of the salinity, along‐channel currents, and TEF at the full model resolution is discussed.

### 3.1 Strait of Juan de Fuca

**Figure 3:**
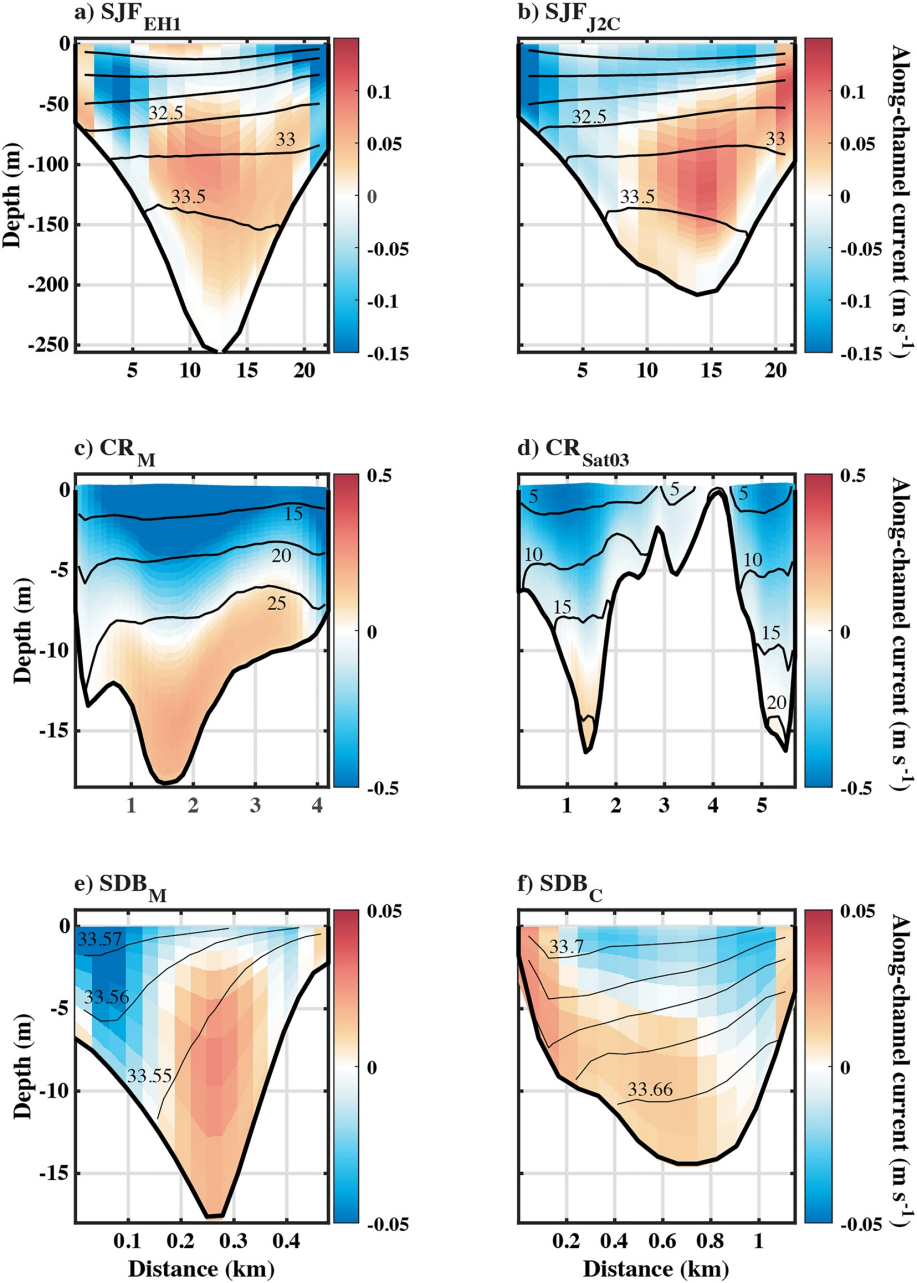
Cross‐sections of annual‐mean fields, taken between the first and last spring high‐tide of the year to avoid tidal aliasing. Shading is along‐channel currents, with positive values (warm colors) indicating flow into the estuary. Contours are isohalines. Note the axes, isohalines, and color scales on each subplot are different.

**Figure 4:**
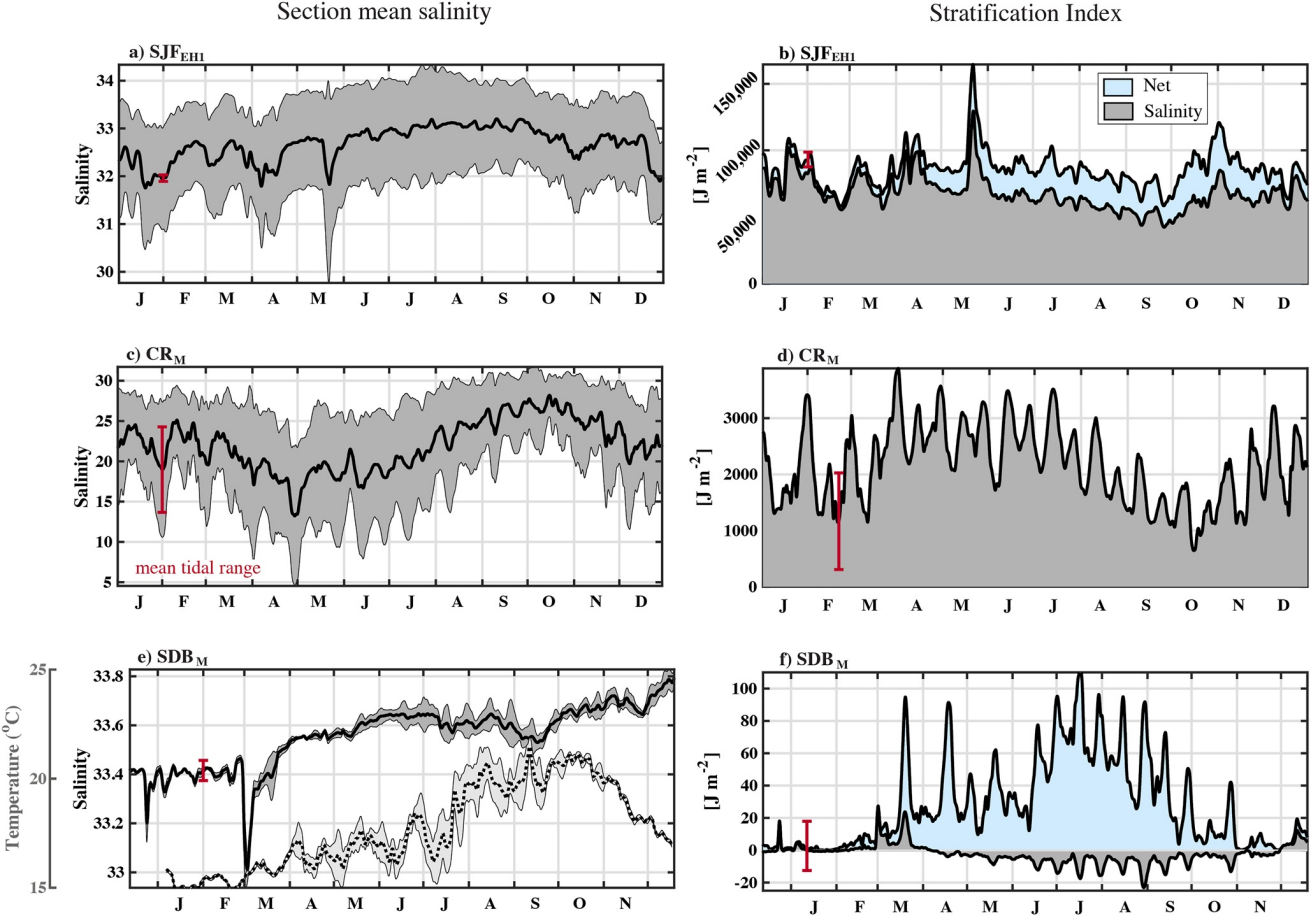
Subtidal salinity variability spatially averaged across sections (a) SJF_EH1_, (c) CR_M_, and (e) SDB_M_. Gray shading is the standard deviation of the spatial mean. In (e), temperature variability is also shown as a dashed line for SDB_M_, where temperature can dominate the stratification. Right panels show the stratification index *ϕ* at (b) SJF_EH1_, (d) CR_M_, and (f) SBD_M_. Gray shading is the stratification due to salinity, while blue shading is the net stratification index. The red vertical bar on each plot shows the mean range of these values over a tidal cycle.

**Figure 5:**
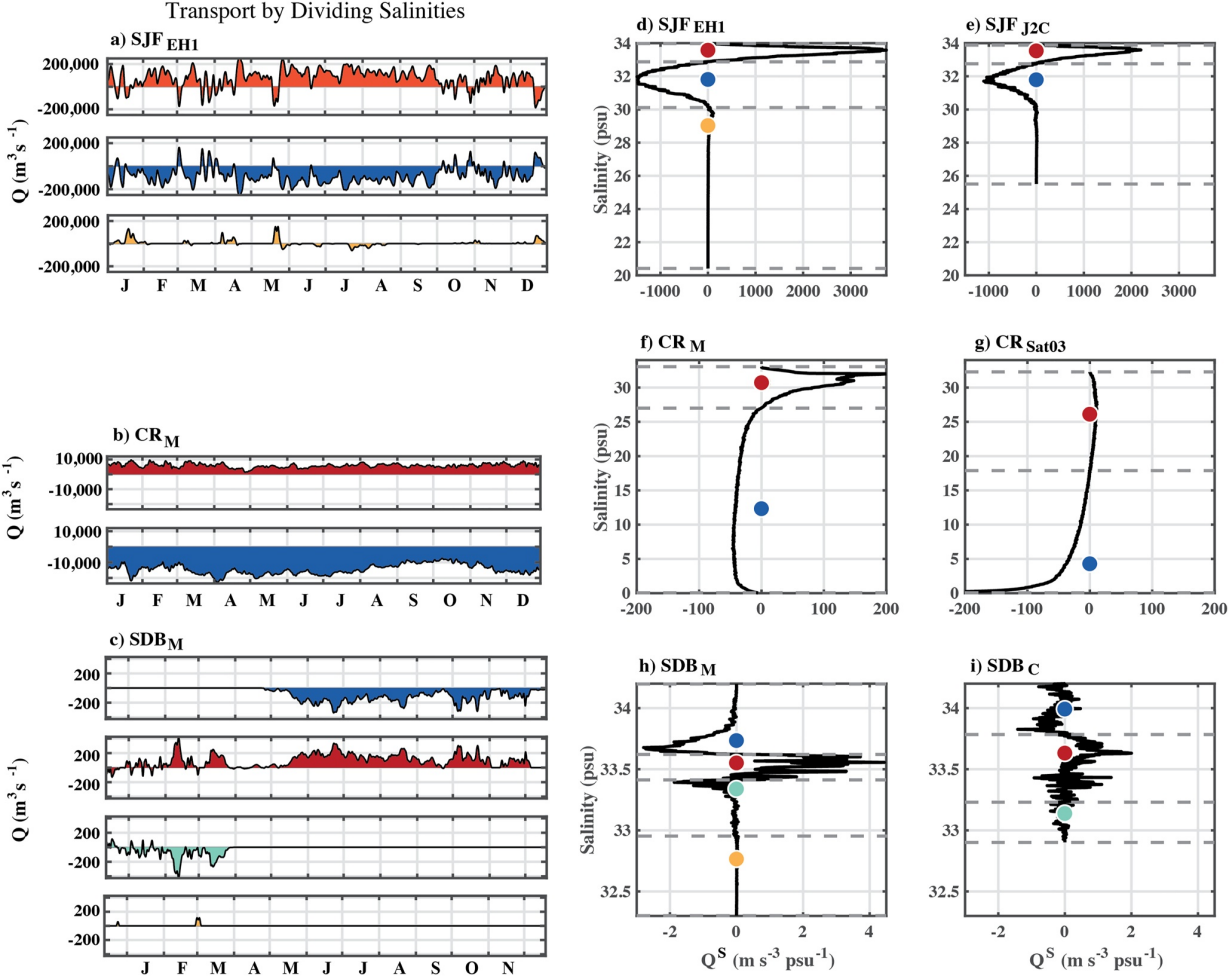
The profile of annual‐mean salinity exchange across each model section. Panels a–c show depth‐integrated magnitudes over time while d‐i show the time‐mean variability over depth. Dashed lines mark the dividing salinities between inflow and outflow layers and colored dots indicate *S*_in_ (red and orange to distinguish inflow layers) and *S*_out_ (blue and cyan to distinguish outflow layers) values. Note that the exchange threshold used to calculate the transport in each layer following Equation 3 is *Q*_thresh_ = 0.01, except as shown for cross‐section SDB_C_ (i), where for the same threshold there were 18 dividing salinities. For SDB_C_ shown here *Q*_thresh_ is 0.1.

Annual mean currents and salinity in the Strait of Juan de Fuca generally exhibit a classical pattern of estuarine circulation with outflow and relatively fresher water near the surface as well as inflow and saltier water at depth (Figure 3a). Occasional intrusions of the Columbia River plume during prolonged downwelling‐favorable winds (Giddings & MacCready, 2017; Hickey et al., 2009) were apparent in the mean currents as an upstream flow near the surface at SJF_EH1_ (Figure 3a); there was also a small, intermittent intrusion of fresher coastal water (<30 psu), associated with increased horizontal shear (Giddings & MacCready, 2017). This fresh surface inflow was not evident in the mean currents at SJF_J2C_ (Figure 3b), which is further from the mouth. Neither the salinity (Figure 4a) nor stratification (Figure 4b) had strong subtidal variability, although temperature had a greater contribution to the vertical stratification in the latter months of the year than in the early spring. Annual mean TEF was predominantly two‐layered, with outflow of fresher water and inflow of relatively saltier water (Figures 5d and 5e) with an occasional third inflow layer at SJF_EH1_ (Figures 5a and 5d).

### 3.2 Columbia River

At CR_M_ near the Columbia River mouth there was also a mean outflow of fresher water near the surface and saltier near‐bottom inflow (Figure 3c). The bathymetry is more complicated at CR_Sat03_ where a shallow sill bisects the channel. At CR_Sat03_ the mean inflow is weak and the mean currents are mostly out of the estuary (Figure 3d). At CR_M_ there was a seasonal cycle in the salinity with a half‐amplitude of 5 psu, similar to the mean tidal range (the salinity time series was low‐pass filtered using a Godin filter; the scale of tidal variability is indicated in red). The stratification at CR_M_ had large variability (similar to the mean) at seasonal, spring‐neap, and higher tidal frequencies (Figure 4d). Note that stratification is calculated from salinity only. The salinity range of the inflow *S*_in_ was greater at CR_Sat03_ than at CR_M_, with more evenly distributed transport across the salinity range, while the outflow across CR_Sat03_ was predominantly at salinities <10 psu. TEF was two‐layered with outflow of fresher water and inflow of relatively saltier water (Figures 5f and 5g). At both sections the outflow *Q*_out_ had greater seasonal variability than the inflow *Q*_in_ (Figure 5b).

### 3.3 San Diego Bay

In San Diego Bay, the annual mean exchange was out of the estuary near the surface, but the surface waters are saltier than at the bottom (Figures 3e and 3f). In this shallow system with relatively little rainfall, vertical stratification at the mouth is thermally controlled much of the year (Chadwick et al., 1996) and varies at seasonal and spring‐neap tidal frequencies (e.g., Figure 4f). Also, the spatial standard deviation of salinity across the section SDB_M_ was small relative to the fluctuation of the mean. Variability in this system is driven by surface heating and evaporation as well as tidal advection and river discharge (Largier, 1995; Largier et al., 1997). TEF was only examined in isohaline coordinates for this study although an analysis in joint temperature‐salinity space is also possible and may be valuable for a dynamical study (Lorenz et al., 2020). The seasonal variability in the exchange at SDB_M_ near the mouth was reflected in the results. In winter the inflow salinity *S*_in_ was <0.3 psu saltier than *S*_out_ (Figure 5h). When the stratification was thermally dominated, the isohaline coordinate TEF reversed and the inflow was fresher than the outflow, again by a small margin. This pattern was similar further upstream at SDB_C_, but at SDB_C_ the magnitude of the exchange, was weaker and to identify three distinct layers of the exchange flow as shown in Figure 5i, the threshold used to calculate the dividing salinities was adjusted to *Q*_percent_ = 0.1, instead of 0.01. For consistency in the following sections, the same value of *Q*_percent_ = 0.01 was used for all of the simulations. The time series of *TEF* parameters *Q*_in_ and *S*_in_ (*Q*_out_ and *S*_out_) were summed (and averaged, following Equation 8) across all inflow (outflow) layers and were not sensitive to *Q*_thresh_.

### 3.4 Horizontal and Vertical Shear

The degree of horizontal shear relative to vertical shear is likely important for estimating how many moorings and sample depths would be needed to capture the exchange flow. At SJF_EH1_ and SJF_J2C_ the channel is wide enough that the Coriolis force can be important (*K*_*e*_ ∼ 2, Table 2) as can be seen in the tilted isopycnals at SJF_J2C_ (Figure 3b). A detailed analysis of the mechanisms driving TEF at SJF_EH1_ and SJF_J2C_ found that the flow was in geostrophic balance, but also that temporal changes were driven by the baroclinic pressure gradient and advection (Giddings & MacCready, 2017). In the Columbia River, the low *Ke* < 2 suggests that horizontal shear is likely to be small relative to vertical shear (Valle‐Levinson, 2008). However, at CR_Sat03_ the steep bathymetry that divides the flow between two channels contributes to horizontal shear. While the role of friction is expected to be stronger in the shallow San Diego Bay, *E*_*k*_ approaches the moderate frictional regime where both horizontal and vertical shear can be found (Valle‐Levinson, 2008). Channel curvature (Figure 1) may also contribute to the observed across‐channel shear and salinity gradients (Figures 3e and 3f). Overall, all of the cross‐sections presented here exhibit both horizontal and vertical shear.

## 4 Evenly Distributed Moorings, TEF_*MN*_

**Figure 6:**
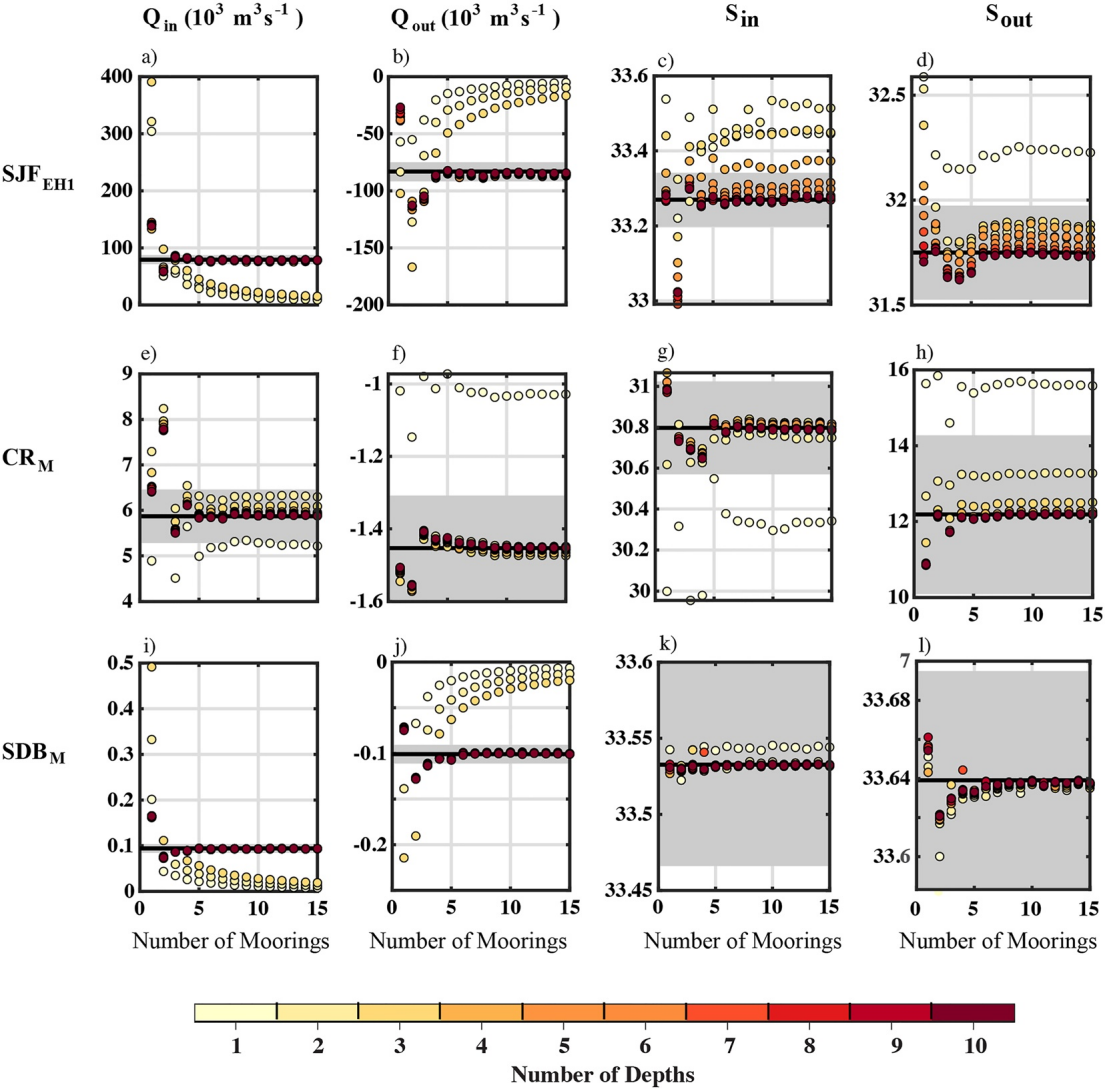
TEF_*MN*_ parameters calculated for *m* = 1–15 evenly distributed moorings across the channel and *n* = 1–10 depths evenly distributed in the vertical direction along each mooring at sections (a–d) SJF_EH1_, (e–h) CR_M_, and (i–l) SDB_M_. Colors indicate the number of depths at each mooring. Black lines and gray shading indicate the magnitude of each TEF_*IJ*_ parameter and the ±10% range (calculated using the freshwater fraction for salinities), respectively.

**Figure 7:**
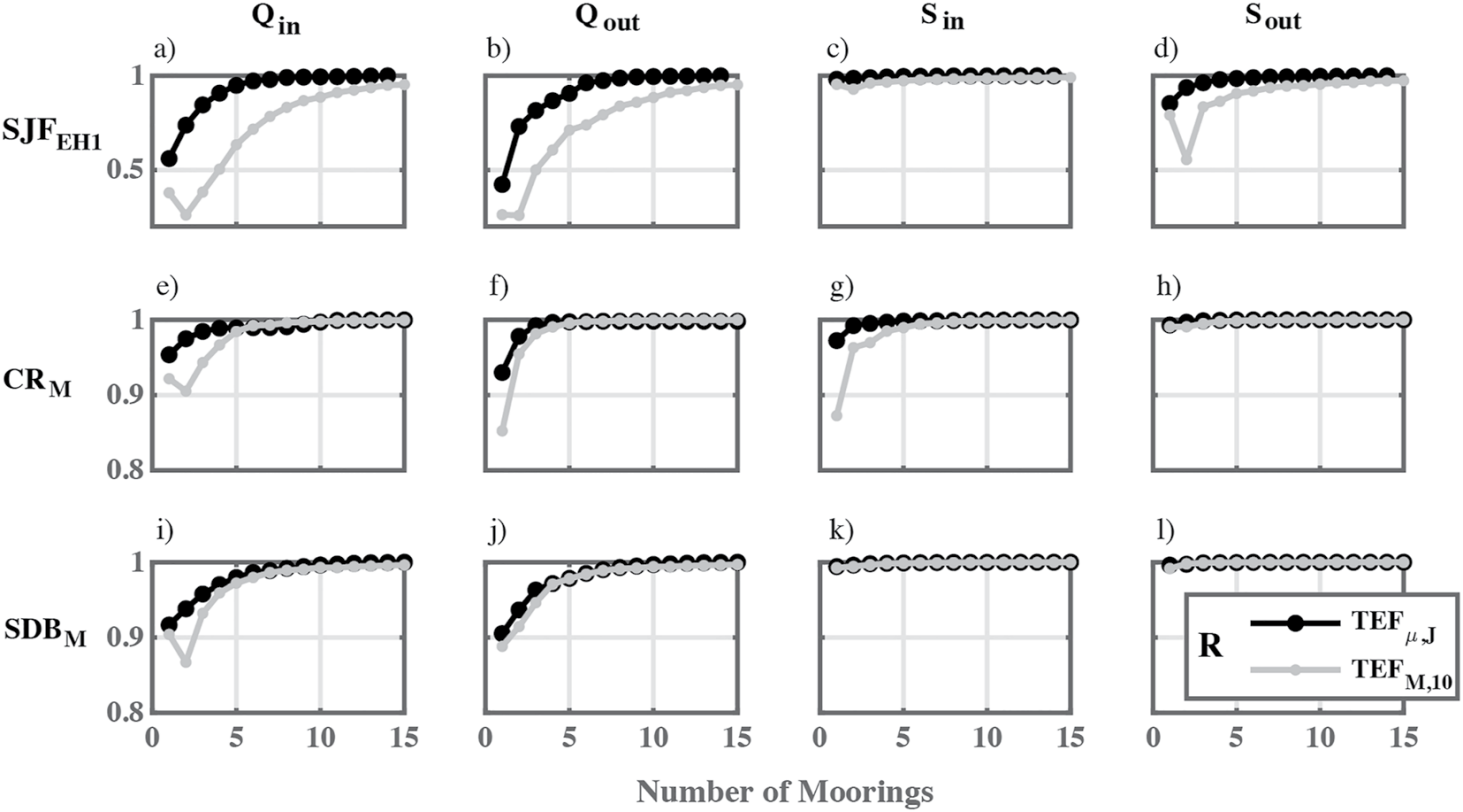
The correlation coefficient between time series of parameters calculated from the full model resolution TEF_*IJ*_ compared to strategically placed moorings TEF_*μJ*_ (black) and evenly distributed moorings TEF_*MN*_ (gray; *N* = 10) as the number of moorings is increased.

TEF_*MN*_ parameters *Q*_in,out_ converged toward TEF_*IJ*_ parameters *Q*_in,out_ as the number of moorings *M* increased as long as there was a minimum number of sample depths *N* at each mooring (Figure 6). The minimum threshold of sample depths for convergence between TEF_*MN*_ and TEF_*IJ*_ was *N* ≤ 4 in most cases (Table 3). Sampling the model fields at fewer depths resulted in smaller *Q*_in,out_ magnitudes. For a small number of sample depths, for example, *N* < 4, TEF_*MN*_ diverged from TEF_*IJ*_ and approached 0 as the number of moorings *M* increased. At sections SJF_EH1_ and SDB_M_ *Q*_in,out_ depended on whether the channel center was sampled; the magnitudes were overestimated if only a single centered mooring was sampled and were underestimated if only two moorings were sampled (i.e., not sampling the channel center; Figure 6). Similarly, there was a lower correlation between *Q*_in,out_ from TEF_*IJ*_ and TEF_*MN*_ for *M* = 2 than for *M* = 1 (Figure 7).

**Table 3:** For Each Parameter (*M*, *N*) Are the Minimum Number of Evenly Distributed Moorings (*M*) and the Minimum Number of Depth Samples (*N*) for Which the Magnitude of TEF_*MN*_ Parameters Converge Toward the TEF_*IJ*_ Values Calculated at the Full Model Resolution

Run	*Q*_in_ *M*	*Q*_out_ *M*	*S*_in_ *M*	*S*_out_ *M*	*Q*_in_ *N*	*Q*_out_ *N*	*S*_in_ *N*	*S*_out_ *N*
SJF (SJF_EH1_)	3	4	3^a^	2^b^	4	4	5	2
SJF (SJF_J2C_)	4^c^	6^d^	3	1	4	4	8	3
CR (CR_M_)	3	1	1	1	2	2	2	2
CR (CR_Sat03_)	7^e^	3	6^f^	1	3	1	1	1
SDB (SDB_M_)	3	4	1	1	4	4	1	1
SDB (SDB_C_)	3	7	1	3^g^	4	4	1	1

*Note*. Convergence is defined here by *Q*_in,out_ and the freshwater fraction equivalent of *S*_in,out_ consistently reaching within 10% of the full model value. These estimates of *M* are conservative and in some cases *M* can be lower such as with a greater number of sample depths *N* (as indicated in each footnote).

^a^ For *N* ≥ 5, *S*_in_ also converges for *M* = 1, but not *M* = 2.

^b^ For *N* ≥ 6, *S*_out_ also converges for *M* = 1.

^c^ For *N* ≥ 4, *Q*_in_ converges for *M* = 3, but not *M* = 4.

^d^ *Q*_out_ also converges with as few as *M* = 4 moorings for *N* ≥ 7.

^e^ For *N* ≥ 3, *Q*_in_ also converges for *M* = 5, but not *M* = 6.

^f^ *S*_in_ also converges for *M* = 3.

^g^ *S*_out_ also converges for *M* = 1, but not *M* = 2.

The deviations of *S*_in,out_ from TEF_*IJ*_ values were relatively small (Figure 6)—within 10% of the freshwater fraction even with only a single mooring, in some cases (Table 3). Particularly at SJF_EH1_ and SJF_J2C_, where there is strong stratification, there was a greater range in *S*_in,out_ as the number of depths *N* varied than as the number of moorings *M* varied.

If too few depths were sampled the annual mean magnitude of *Q*_in,out_ and *S*_in,out_ calculated from TEF_*MN*_ did not converge to TEF_*IJ*_, but the number of sample depths required for convergence did not change as *M* increased (e.g., Figure 6a). In most cases the TEF_*MN*_ parameters *S*_in,out_ converged toward TEF_*IJ*_ parameters at values of *M* and *N* that were similar to or smaller than the number of moorings and sample depths over which *Q*_in,out_ converged. Thus, *Q*_in,out_ were the limiting parameters with the TEF_*MN*_ method.

## 5 Strategically Distributed Moorings, TEF_*μJ*_

In order to assess the sensitivity of TEF on sampling method, a strategic approach to lateral mooring placement rather than a geometric distribution was tested. The strategic approach, TEF_*μJ*_, incrementally added moorings that contributed the maximum correlation improvement between the sampled parameters, TEF_*μJ*_ and TEF_*IJ*_. Due to the iterative nature of this method, only variability in the lateral mooring placement was assessed, and the water column was sampled at the full vertical model resolution, *N* = *J*. While the maximum correlation for a given number of moorings was slightly less with fewer vertical samples (*N* < *J*), the patterns were similar to those presented here for *N* = *J*, particularly for *N* ≥ 4.

Using the time series correlation to strategically select lateral mooring locations, the correlation between the sub‐sampled TEF_*μJ*_ and TEF_*IJ*_ parameters converged to 1 for fewer moorings than TEF_*MN*_ parameters (Figure 7). However, this distinction is minimal for the cross‐sections in the Columbia River and San Diego Bay (e.g., Figures 7e and 7l) where the correlation is high (>0.8) even when only one or two moorings were used to sample the exchange flow. The high correlation between TEF_*IJ*_ and that from a single optimal mooring (i.e., TEF_*μ* = 1,*J*_) in the CR and SDB is likely due to the temporal variability in the exchange flow having a wide spatial signal, that is, similar temporal variability over the full cross‐section. This is opposed to SJF where temporal variations in exchange have a strong spatial signature, such as those associated with intermittent downwelling‐favorable winds (e.g., Figure 3a and Giddings & MacCready, 2017).

**Figure 8:**
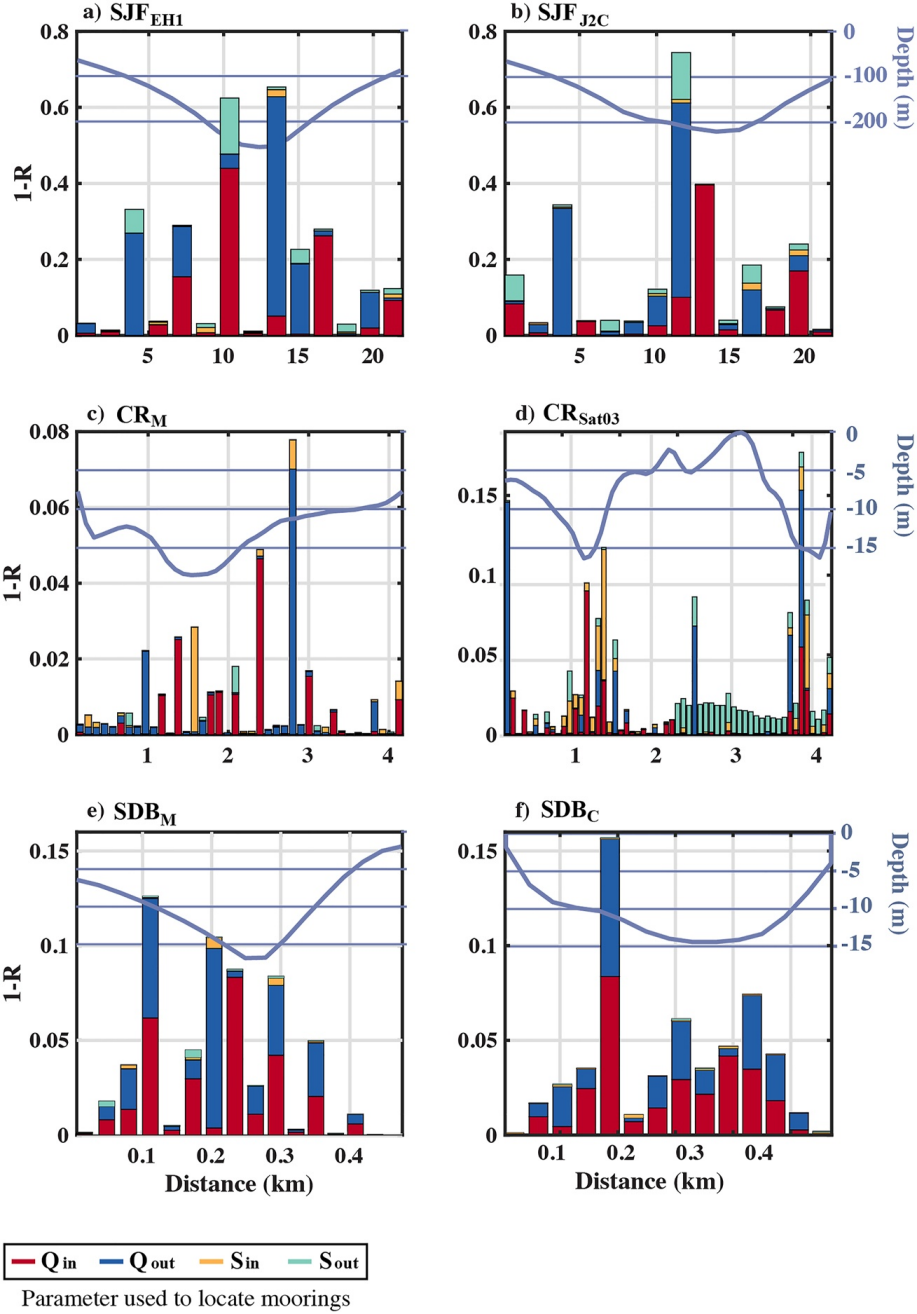
The improvement in the time series correlation between TEF_*μJ*_ and TEF_*IJ*_ realized from adding each additional mooring *μ*. The horizontal axis marks the location of each mooring along the cross‐section (bathymetry is overlain in black on the right axes). Colors indicate different parameters *Q*_in_ , *Q*_out_, *S*_in_, and *S*_out_.

The mooring order varied based on which TEF parameter they were designed to capture (i.e., *Q*_in_, *Q*_out_, *S*_in_ or *S*_out_; Figure 8). However, the placement of the first mooring tended to be in deeper parts of the channel. In general the strategic mooring placement spanned the section. If each cross‐section was geometrically divided into thirds, the first triad of moorings (i.e., the three tallest bars of each color on Figure 8) was roughly distributed across those three sections, resulting in a sampling distribution that was similar between *TEF*_*μ* = 3,*J*_ and *TEF*_*M* = 3,*N*_. Despite this similarity, and despite the correlation converging more quickly for strategic moorings (Figure 7), the minimum number of moorings *μ* before *Q*_in,out_ converged to within 10% of TEF_*IJ*_ values was greater for the strategic sampling approach (Table 4) than when using the evenly spaced sampling approach (Table 3). This implies that the areas of high TEF variance do not fully correspond with the maximum TEF magnitude.

**Table 4:** The Minimum Number of Strategically Distributed Moorings for Which the Magnitude of TEF_*μJ*_ Parameters *Q*_in,out_ and *S*_in,out_ Converge Toward the TEF_*IJ*_ Values Calculated at the Full Model Resolution

Run	*Q*_in_ *μ*	*Q*_out_ *μ*	*S*_in_ *μ*	*S*_out_ *μ*
SJF (SJF_EH1_)	4	9	1	1
SJF (SJF_J2C_)	6	2	3	1
CR (CR_M_)	10	3	2	1
CR (CR_Sat03_)	16	2	1	1
SDB (SDB_M_)	8	1	1	1
SDB (SDB_C_)	7	13	1	1

*Note*. Convergence is defined here by *Q*_in,out_ and the freshwater fraction equivalent of *S*_in,out_ reaching within 10% of the full model value.

**Figure 9:**
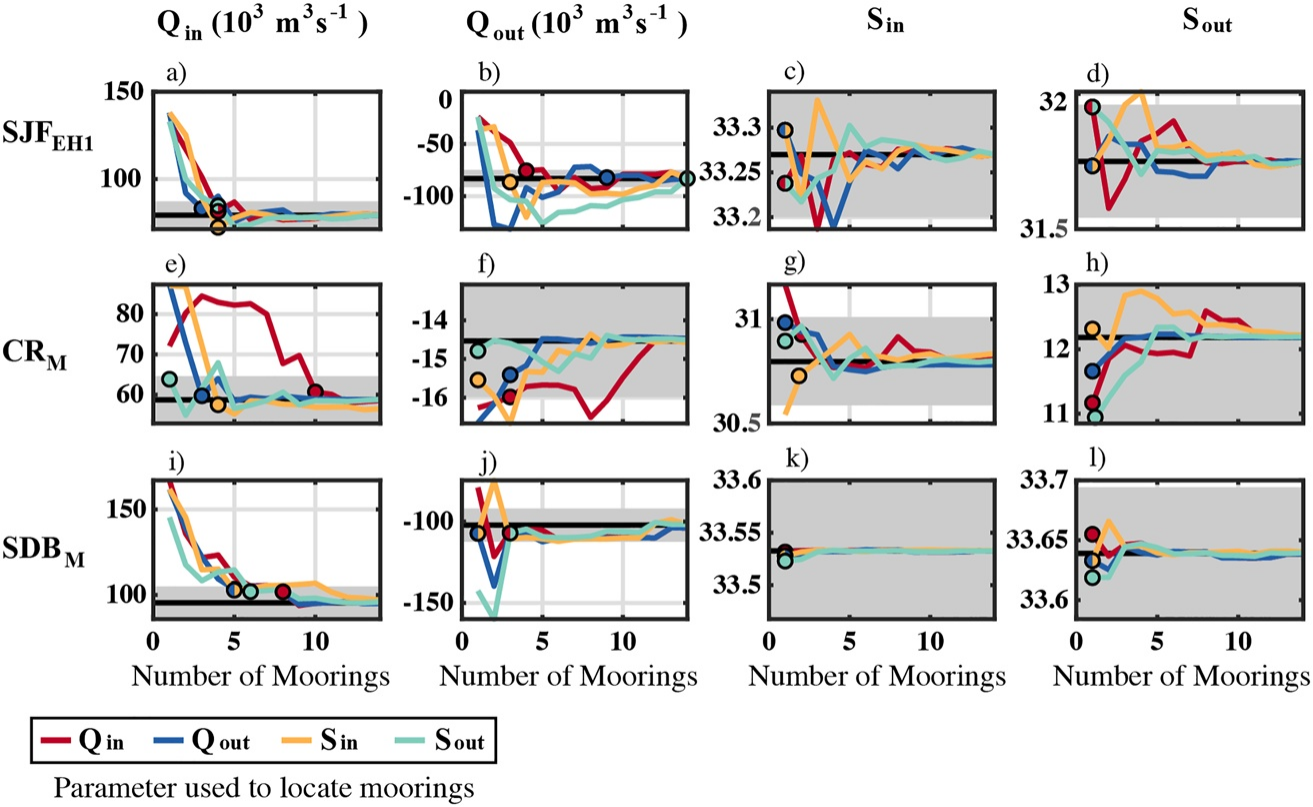
TEF_*μJ*_ parameters calculated for *μ* = 1–15 moorings strategically placed across the channel, using the full vertical grid resolution at each mooring. Black lines and gray shading indicate the magnitude of each TEF_*IJ*_ parameter and the ±10% range (calculated using the freshwater fraction for salinities), respectively. Colored lines indicate the parameter used to determine the order of mooring placement by maximizing the correlation between TEF_*μJ*_ and TEF_*IJ*_. Colored dots mark the first point where each TEF_*μJ*_ parameter converges to within 10% of the corresponding TEF_*IJ*_ parameter (e.g., Table 4).

This investigation of TEF_*μJ*_ provided insight into the sensitivity of the results to the specific sample locations by comparing the variation in results using the mooring order from each TEF_*μJ*_ parameter (Figure 9). The results presented in Figures 7 and 8 and throughout the text primarily focus on the outcomes of each parameter of TEF_*μJ*_ with strategic moorings selected based on that same parameter. This means that the single mooring (*μ* = 1) with the highest correlation to TEF_*IJ*_ for the parameter *Q*_in_ is not necessarily the same mooring location with the highest correlation to TEF_*IJ*_ for the parameters *Q*_out_, *S*_in_, or *S*_out_. Similarly, the second mooring (*μ* = 2) is not necessarily the same across parameters *Q*_in_, *Q*_out_, *S*_in_, or *S*_out_ and so on. For example, there was some variation in the pattern of *Q*_in_ for increasing *μ*, between the mooring order strategically determined using *Q*_in_ compared to the mooring order strategically determined using *Q*_out_, *S*_in_, or *S*_out_ (Figure 9a). As the number of moorings increased, the magnitude of *Q*_in,out_ and *S*_in,out_ converged toward the TEF_*IJ*_ results. In most cases the rate of convergence was qualitatively similar regardless of which TEF parameter was used to determine the mooring order, with some variation in the point at which convergence within 10% of TEF_*IJ*_ was reached (Figure 9). One exception that stood out was that the number of moorings (*μ*) at CR_M_ before *Q*_in_ converged to within 10% of the TEF_*IJ*_ value was more than double the minimum number of moorings (*μ*) to reach the same threshold when the mooring placement was optimized for *Q*_out_, *S*_in_, and *S*_out_ (Figure 9e). It is unclear why the mooring placement for *Q*_in_ was particularly inefficient for capturing the magnitude of *Q*_in_, but individual components of the exchange flow and salt flux can exhibit different spatial patterns and timescales of variability (e.g., Lerczak et al., 2006), which likely contributed to a misalignment between the locations with the most variance and those with the greatest magnitude in the exchange flow.

## 6 Case Studies: Hypothetical Mooring Deployments, TEF_case_

Both evenly and strategically distributed mooring approaches tested here are skewed toward numerical modeling applications because of the sampling resolution, which would require many sensors and mooring lines that extend across nearly the full water column. In particular, strategic mooring placement requires extensive a priori knowledge of the system. In an effort to connect this analysis more closely to observations, a specific case study, TEF_case_, was also examined as described in Section 2.4.4.

**Figure 10:**
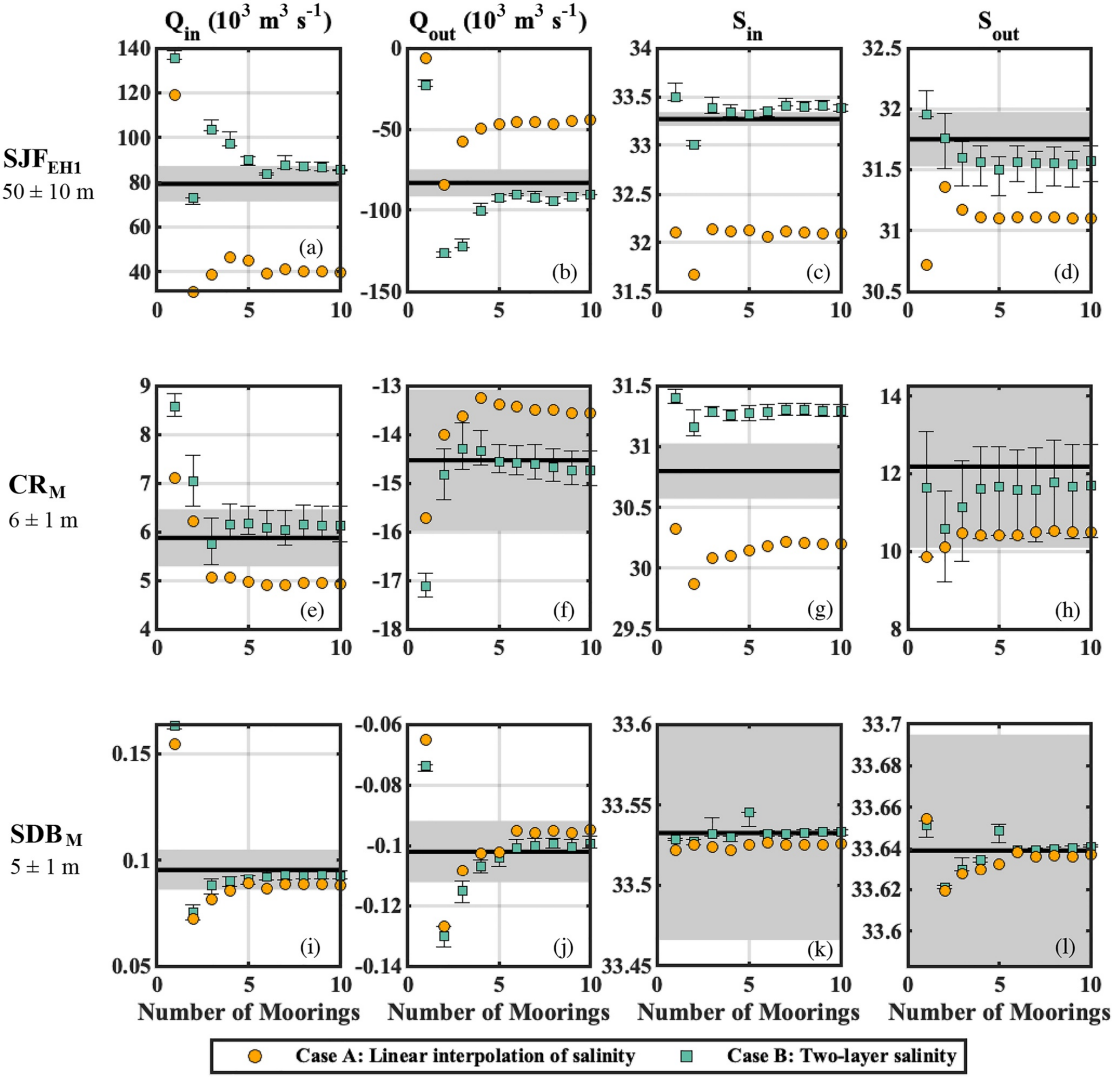
Case study parameters calculated for *m* = 1–10 evenly distributed moorings across the channel with salinity sampled 1 m above the seafloor and below sea level and velocity sampled at the full model vertical resolution. Results are shown from sections (a–d) SJF_EH1_, (e–h) CR_M_, and (i–l) SDB_M_. Two methods of interpolating salinity values to the velocity sampling points along each mooring line are shown: linear interpolation (orange circles) and assuming two mixed layers of uniform salinity with the interface at a fixed depth (green squares). Squares mark the TEF_case_ 2‐layer parameter values using a fixed interface depth (50, 6, and 5 m at SJF_EH1_, CR_M_, and SDB_M_, respectively) and vertical bars mark the range of values if the interface depth is varied by a fixed amount for each section (±10, ±1, and ±1 m at SJF_EH1_, CR_M_, and SDB_M_, respectively). Black lines and gray shading indicate the magnitude of each TEF_*IJ*_ parameter and the ±10% range (calculated using the freshwater fraction for salinities), respectively.

The salinity interpolation impacted both the calculated exchange volume transport and salinities (Figure 10). Similar to the other sampling methods presented, as the number of moorings *M* increased, TEF_case_ converged to a similar value for *M* ≥ 4, although not necessarily toward TEF_*IJ*_. At SDB_M_, where salinity stratification is weak, TEF_case_ converged within ≈10% of TEF_*IJ*_ with ≥4 moorings. In other words, the vertical salinity interpolation method did not matter. At sections SJF_EH1_ and CR_M_, however, the results were mixed with the two‐layer approximation generally performing better than the linear interpolation with varying sensitivity.

At SJF_EH1_, linearly interpolating salinity led to underestimating the magnitude of the exchange volume transport (Figures 10a and 10b). This is possible when opposing currents are classified into the same salinity range, which reduces the net exchange magnitude within that salinity class. In this case, a two‐layer *S* approximation led to values of TEF_case_ closer to TEF_*IJ*_ than a linear interpolation. The results were fairly insensitive to the interface depth between the two uniform salinity layers, except for *S*_out_.

At CR_M_, the two‐layer *S* approximation performed slightly better than the linear interpolation (Figures 10c–10h). Whether the two‐layer approximation resulted in an improvement of *Q*_in,out_ over the linear approximation was sensitive to the interface depth. In neither case did the TEF_case_ exchange salinity *S*_in_ converge to within 10% of the full TEF_*IJ*_ value.

## 7 Discussion

The results of this study suggest that TEF via *in situ* moorings can be well approximated in many situations with ≤4 moorings across a channel. However, there are some limitations to this analysis that may constrain the generalization of these results. The magnitude and structure of the exchange flow for each estuary may depend on the specific thresholds, time periods, and cross‐sections. The sampling approaches explored here were limited to a small number of relatively simple designs and were not adapted for the particular bathymetry or oceanic conditions at each cross‐section. Also the examples chosen, while spanning significant parameter space in terms of estuary type and geometry, were not exhaustive. The practical applicability of the results presented in this study are discussed in the following section in the context of these limitations.

The magnitude and structure of the exchange flow for each model may depend on specific thresholds, time periods, and cross‐sections chosen for the calculation. The TEF approach requires the number of salinity bins *N*_bins_ and a threshold for identifying the cut‐off between dividing salinities *Q*_thresh_ to be defined. While the dividing salinity approach (Lorenz et al., 2019) reduces the sensitivity to these choices (here, *N*_bins_ = 500 and *Q*_thresh_ = 0.01), in the weakly stratified San Diego Bay which experiences large variability on tidal time scales (e.g., Figure 4f) the number and location of dividing salinities varied depending on threshold choices (e.g., Figure 5i). However the results presented here were summed over the incoming and outgoing layers and were insensitive to these choices, even at section SDB_C_. At the other cross‐sections, the results were insensitive to variations in *N*_bins_ and *Q*_thresh_. Another consideration is that the exchange flow can vary over time, with sampling frequency, and with model grid resolution (Figures 5a–5c) such that the TEF magnitude may be specific to the particular periods examined (in particular annual vs. seasonal time periods). The conclusions drawn from this analysis focus on comparing the relative change in the TEF across methods with each method applied over the same time period and model cross‐section to reduce the dependence on the specific time period and resolution. However, if only part of the year was examined when surface intrusions were absent at SJF_EH1_, for example, the optimal lateral spacing may have been impacted.

Variances in the TEF parameter time series were strongly correlated between methods (*R* > 0.85) even with a single mooring, except at SJF_EH1_ (Figure 7). The high correlation is likely due to the strong spring‐neap and seasonal variability at CR_M_ and SDB_M_ (Figure 5) that are spatially coherent as opposed to the seasonal spatial variability caused by surface intrusions at SJF_EH1_. That a single mooring location captures most of the TEF variance also suggests that optimizing the correlation may not be a useful method for strategic mooring placement to measure exchange flows. Even at SJF_EH1_ where the variance in the exchange flow was improved using the TEF_*μJ*_ method relative to TEF_*MN*_ (Figures 7a and 7b), the minimum number of moorings for which the TEF_*μJ*_ magnitude converged to within 10% of TEF_*IJ*_ was greater than the minimum number of geometrically distributed moorings (Table 3 compared to 4). Also, at CR_M_, 10 mooring samples were required for *Q*_in_ using the TEF_*μJ*_ method to converge to within 10% of TEF_*IJ*_ result (Table 4). These results suggest that the locations across each section with the most temporal variance are not the same locations where the majority of the transport occurs. That in most cases 4 evenly distributed moorings captured >90% of the exchange flow (Table 3) and most of the flow variance (Figure 7) also suggests that a straightforward sampling plan—as long as there are sufficient number of moorings and depths sampled—is likely to capture both features (variance and magnitude) of the exchange. This is also supported by the observation that there was generally little difference in the results as mooring placement varied (Figure 9).

While the selected cross‐sections span a bay, salt‐wedge, and fjord estuary type with differing scales and geometric complexity (Geyer & MacCready, 2014), this subset does not comprehensively cover the full range of estuarine shapes, sizes, and dynamics. Nevertheless, given the range investigated here, the similarity of the results was striking. First, using only a single mooring led to an over‐estimate of *Q*_in_ in every case. This over‐estimate could be several times the magnitude of *Q*_in_ from the full model resolution. In particular for the transects sampled in SJF and SDB, with relatively simple U‐shaped channels, using only two laterally distributed moorings that did not sample the channel center led to an underestimate of *Q*_in_ (e.g., Figures 6a and 6i). While the greatest inflow tended to be concentrated toward the central and deeper channel, the lateral distribution of the outflow was more variable (Figure 3). Second, it was encouraging—from the perspective of capturing exchange flows in a range of systems with limited a priori knowledge—that using evenly distributed moorings performed as well or better than the strategic sampling strategy (Table 3 compared to Table 4) and also that the results converged toward *TEF*_*IJ*_ even as the specific sampling locations were varied (Figure 9). The mean number of evenly distributed lateral mooring locations across each channel to resolve (*Q*_in,out_) to within 10% was *M* = 4.0 ± 1.8 with *N* = 3.3 ± 1.1 sample depths evenly distributed across the water column and *M* = 2.0 ± 1.5, *N* = 2.3 ± 2.1 to resolve *S*_in,out_ (Table 3).

Further studies of systems dominated by either horizontal or vertical shear would be needed to assess if the sampling resolution might be related to *K*_*e*_ and *E*_*k*_. While *K*_*e*_ and *E*_*k*_ are comparable across systems, the predictive utility of such parameters may be complicated by channel curvature and variable bathymetry and requires a priori knowledge of the flow. However, the degree of stratification did appear to be important to understand how the vertical and horizontal sampling resolution impacted TEF. At SJF_EH1_ and CR_M_, TEF_*MN*_ converged to within 10% of TEF_*IJ*_ with ≤4 evenly distributed moorings (Figure 6) while TEF_case_ did not always converge to the same values as TEF_*IJ*_ (Figure 10). This suggests that when stratification is important more vertical resolution of *S* reduces the number of moorings needed across the channel. Also, at these stratified sections the result depends on the vertical interpolation of *S*. In contrast, at SDB_M_ the number of moorings needed to accurately calculate TEF_*MN*_ (Figure 6) was similar to TEF_case_ (Figure 10) and was similar between cases A and B. At SDB_M_ with *u* and *S* samples evenly distributed vertically the exchange volume flux only converged for *N* ≥ 4, while in the case studies the TEF_case_ exchange volume fluxes converged to TEF_*IJ*_ when *S* was sampled at only two depths, near‐surface and near‐bottom.

Given that only three estuaries (6 cross‐sections) were examined here and that this study utilized numerical model output, the question remains: how realistic would it be to apply the results of these experiments to observations and in other systems? One general limitation of observational studies is the cost of moorings (anchors, line, floats, etc.) as well as of the individual sensors. The results of this study may be useful to constrain an estimate of the sign and possibly the magnitude of the error for sampling studies with fewer moorings. For example, one could extrapolate that a single mooring centered in a deep part of the channel is likely to overestimate the magnitude of the exchange volume transport. The outcome of the case studies also suggest that the salinity does not have to be sampled at the same resolution as the currents to estimate the exchange flow with relatively high accuracy, although as stratification increases, identifying the best salinity interpolation remains a challenge (Aristizábal & Chant, 2015). In channels with high ship traffic, near‐surface measurements can be particularly challenging. However, it may be encouraging that the results here demonstrated relatively little sensitivity to the specific sample location, that is, one could place a mooring outside of a navigational channel.

## 8 Conclusions

Exchange between estuaries and the coastal ocean or through inland seas is an important driver of the circulation, mixing, biology, and chemistry on both ends of the exchange. Significant progress has been made in calculating this exchange in estuarine conditions with time‐varying stratification and flow, strong vertical and lateral velocity shear and salinity gradients, and complex bathymetry using the TEF method (Lorenz et al., 2019, 2020). However, application of this theory has predominantly utilized numerical models where the salinity and velocity are highly resolved in space and time. In this analysis TEF was calculated using various methods to sub‐sample realistic numerical models in order to understand the sensitivity of TEF and to develop recommendations for minimal sampling thresholds that accurately reproduce the exchange flow. Three different estuaries were examined, including San Diego Bay, the Columbia River, and the Salish Sea exchange through the Strait of Juan de Fuca. These examples span a range of estuary types (bay, salt‐wedge, and fjord, respectively), scales, depths, and channel bathymetries. Evenly distributed sample locations across the channel, representative of moorings, was the most efficient way to capture the TEF. Three to four moorings were typically the minimum lateral sample distribution required to capture ≥90% of the exchange transport rate *Q*_in_ and *Q*_out_. In most cases, the exchange volume transport was the limiting parameter, requiring more moorings to measure than the exchange flow salinities *S*_in_ and *S*_out_. The minimum vertical resolution to capture ≥90% of the TEF was similar, *N* ≥ 4, and was also limited by *Q*_in_ and *Q*_out_. Although the exchange calculated by these methods is also dependent on resolving the salinity, the TEF was less sensitive to resolving salinity at the same vertical resolution as velocity and less sensitive to the salinity interpolation method in systems where there was less vertical stratification (e.g., the San Diego Bay, as compared to the Columbia River or the Strait of Juan de Fuca). The TEF could be reproduced by resolving the currents throughout the water column and only sampling salinity near the surface and bottom. In comparison to geometrically distributing moorings across the channel, strategic sampling based on capturing the temporal exchange flow variance did not improve the ability to capture the exchange flow magnitude, likely a result of the fact that locations of strongest exchange flow are often not the locations with the highest variance. This method also requires a priori knowledge of the flow field, and is ambiguous depending on which aspect of the TEF (i.e., *Q*_in,out_ or *S*_in,out_) is used to calculate the variance and is therefore not a recommended approach for estuary sampling methodology. Overall the results presented here are promising suggesting that TEF can be captured well with a reasonable number of cross‐sectionally distributed moorings and sampling depths and can be used to estimate the sign and magnitude of errors. Future work to examine the exchange flow through a wider range and greater number of estuaries and channels could be useful to further refine the number of moorings and the approach to determine the best salinity interpolation.

## Data Availability

Model cross‐sections and analysis code used in this analysis are hosted at the UCSD Library Digital Collections (https://doi.org/10.6075/J0DJ5FS8).

## References

[jgrc25233-bib-0001] Aristizábal, M. F. , & Chant, R. J. (2015). An observational study of salt fluxes in Delaware Bay. Journal of Geophysical Research: Oceans, 120(4), 2751–2768. 10.1002/2014JC010680

[jgrc25233-bib-0002] Banas, N. S. , MacCready, P. , & Hickey, B. M. (2009). The Columbia River plume as cross‐shelf exporter and along‐coast barrier. Continental Shelf Research, 29(1), 292–301. 10.1016/j.csr.2008.03.011

[jgrc25233-bib-0003] Barron, C. N. , Kara, A. B. , Martin, P. J. , Rhodes, R. C. , & Smedstad, L. F. (2006). Formulation, implementation and examination of vertical coordinate choices in the global navy coastal ocean model (NCOM). Ocean Modelling, 11(3), 347–375. 10.1016/j.ocemod.2005.01.004

[jgrc25233-bib-0004] Becherer, J. , Flöser, G. , Umlauf, L. , & Burchard, H. (2016). Estuarine circulation versus tidal pumping: Sediment transport in a well‐mixed tidal inlet. Journal of Geophysical Research: Oceans, 121(8), 6251–6270. 10.1002/2016JC011640

[jgrc25233-bib-0005] Booij, N. , Ris, R. C. , & Holthuijsen, L. H. (1999). A third‐generation wave model for coastal regions: 1. Model description and validation. Journal of Geophysical Research: Oceans, 104(C4), 7649–7666. 10.1029/98JC02622

[jgrc25233-bib-0006] Boyer, E. W. , Goodale, C. L. , Jaworsk, N. A. , & Howarth, R. W. (2002). Anthropogenic nitrogen sources and relationships to riverine nitrogen export in the northeastern USA. Biogeochemistry, 57(1), 137–169. 10.1023/A:1015709302073

[jgrc25233-bib-0007] Brown, C. , & Ozretich, R. (2009). Coupling between the coastal ocean and Yaquina Bay, Oregon: Importance of oceanic inputs relative to other nitrogen sources. Estuaries and Coasts, 32(2), 219–237. 10.1007/s12237-008-9128-6

[jgrc25233-bib-0008] Burchard, H. , & Badewien, T. H. (2015). Thermohaline residual circulation of the wadden sea. Ocean Dynamics, 65(12), 1717–1730. 10.1007/s10236-015-0895-x

[jgrc25233-bib-0009] Burchard, H. , Bolding, K. , Feistel, R. , Graewe, U. , Klingbeil, K. , MacCready, P. , et al. (2018). The Knudsen theorem and the total exchange flow analysis framework applied to the Baltic Sea. Progress in Oceanography, 165, 268–286. 10.1016/j.pocean.2018.04.004

[jgrc25233-bib-0010] Burchard, H. , & Hetland, R. D. (2010). Quantifying the contributions of tidal straining and gravitational circulation to residual circulation in periodically stratified tidal estuaries. Journal of Physical Oceanography, 40(6), 1243–1262. 10.1175/2010JPO4270.1

[jgrc25233-bib-0011] Chadwick, D. B. , Largier, J. L. , & Cheng, R. T. (1996). The role of thermal stratification in tidal exchange at the mouth of San Diego Bay. In Buoyancy effects on coastal and estuarine dynamics (pp. 155–174). American Geophysical Union (AGU). 10.1029/CE053p0155

[jgrc25233-bib-0012] Chen, S. N. , Geyer, W. R. , Ralston, D. K. , & Lerczak, J. A. (2012). Estuarine exchange flow quantified with isohaline coordinates: Contrasting long and short estuaries. Journal of Physical Oceanography, 42(5), 748–763. 10.1175/JPO-D-11-086.1

[jgrc25233-bib-0013] Cheng, P. , Valle‐Levinson, A. , & Swart, H. E. d. (2011). A numerical study of residual circulation induced by asymmetric tidal mixing in tidally dominated estuaries (Vol. 116). 10.1029/2010JC006137

[jgrc25233-bib-0014] Conroy, T. , Sutherland, D. A. , & Ralston, D. K. (2020). Estuarine exchange flow variability in a seasonal, segmented estuary. Journal of Physical Oceanography, 50(3), 595–613. 10.1175/JPO-D-19-0108.1

[jgrc25233-bib-0015] Davis, K. A. , Banas, N. S. , Giddings, S. N. , Siedlecki, S. A. , MacCready, P. , Lessard, E. J. , et al. (2014). Estuary‐enhanced upwelling of marine nutrients fuels coastal productivity in the. U.S. Pacific northwest. Journal of Geophysical Research: Oceans, 119(12), 8778–8799. 10.1002/2014JC010248

[jgrc25233-bib-0016] Ganju, N. K. , Hayn, M. , Chen, S.‐N. , Howarth, R. W. , Dickhudt, P. J. , Aretxabaleta, A. L. , & Marino, R. (2012). Tidal and groundwater fluxes to a shallow, microtidal estuary: Constraining inputs through field observations and hydrodynamic modeling. Estuaries and Coasts, 35(5), 1285–1298. 10.1007/s12237-012-9515-x

[jgrc25233-bib-0017] Garvine, R. W. (1995). A dynamical system for classifying buoyant coastal discharges. Continental Shelf Research, 15(13), 1585–1596. 10.1016/0278-4343(94)00065-U

[jgrc25233-bib-0018] Geyer, W. R. , & MacCready, P. (2014). The estuarine circulation. In S. H. Davis & P. Moin (Eds.), Annual reviews, Annual review of fluid mechanics (Vol. 46, pp. 175–197). 10.1146/annurev-marine-120308-081015

[jgrc25233-bib-0019] Geyer, W. R. , Ralston, D. K. , & Chen, J.‐L. (2020). Mechanisms of exchange flow in an estuary with a narrow, deep channel and wide, shallow shoals. Journal of Geophysical Research: Oceans, 125(12), e2020JC016092. 10.1029/2020JC016092

[jgrc25233-bib-0020] Giddings, S. N. , & MacCready, P. (2017). Reverse estuarine circulation due to local and remote wind forcing, enhanced by the presence of along‐coast estuaries. Journal of Geophysical Research: Oceans, 122(12), 10184–10205. 10.1002/2016JC012479

[jgrc25233-bib-0021] Giddings, S. N. , MacCready, P. , Hickey, B. M. , Banas, N. S. , Davis, K. A. , Siedlecki, S. A. , et al. (2014). Hindcasts of potential harmful algal bloom transport pathways on the Pacific northwest coast. Journal of Geophysical Research: Oceans, 119(4), 2439–2461. 10.1002/2013JC009622

[jgrc25233-bib-0022] Griffin, D. A. , & LeBlond, P. H. (1990). Estuary/ocean exchange controlled by spring‐neap tidal mixing. Estuarine, Coastal and Shelf Science, 30(3), 275–297. 10.1016/0272-7714(90)90052-S

[jgrc25233-bib-0023] Hickey, B. , McCabe, R. , Geier, S. , Dever, E. , & Kachel, N. (2009). Three interacting freshwater plumes in the northern California current system. Journal of Geophysical Research, 114, C00B03. 10.1029/2008JC004907 PMC2867361 20463844

[jgrc25233-bib-0024] Kara, A. B. , Barron, C. N. , Martin, P. J. , Smedstad, L. F. , & Rhodes, R. C. (2006). Validation of interannual simulations from the 1/8° global navy coastal ocean model (NCOM). Ocean Modelling, 11(3), 376–398. 10.1016/j.ocemod.2005.01.003

[jgrc25233-bib-0025] Karna, T. , & Baptista, A. M. (2016). Evaluation of a long‐term hindcast simulation for the Columbia River estuary. Ocean Modelling, 99, 1–14. 10.1016/j.ocemod.2015.12.007

[jgrc25233-bib-0026] Karna, T. , Baptista, A. M. , Lopez, J. E. , Turner, P. J. , McNeil, C. , & Sanford, T. B. (2015). Numerical modeling of circulation in high‐energy estuaries: A Columbia River estuary benchmark. Ocean Modelling, 88, 54–71. 10.1016/j.ocemod.2015.01.001

[jgrc25233-bib-0027] Kasai, A. , Hill, A. E. , Fujiwara, T. , & Simpson, J. H. (2000). Effect of the Earth's rotation on the circulation in regions of freshwater influence. Journal of Geophysical Research, 105(C7), 16961–16969. 10.1029/2000JC900058

[jgrc25233-bib-0028] Knudsen, M. (1900). In Ein hydrographischer lehrsatz (Vol. 28, pp. 316–320).

[jgrc25233-bib-0029] Kumar, N. , Voulgaris, G. , Warner, J. C. , & Olabarrieta, M. (2012). Implementation of the vortex force formalism in the coupled ocean‐atmosphere‐wave‐sediment transport (COAWST) modeling system for inner shelf and surf zone applications. Ocean Modelling, 47, 65–95. 10.1016/j.ocemod.2012.01.003

[jgrc25233-bib-0030] Largier, J. L. (1995). San Diego Bay circulation, a study of the circulation of water in San Diego Bay for the purpose of assessing, monitoring and managing the transport and potential accumulation of pollutants and sediment in San Diego Bay. Final Report.

[jgrc25233-bib-0031] Largier, J. L. , Hearn, C. J. , & Chadwick, D. B. (1996). Density structures in “low inflow estuaries”. In Buoyancy effects on coastal and estuarine dynamics (pp. 227–241). American Geophysical Union (AGU). 10.1029/CE053p0227

[jgrc25233-bib-0032] Largier, J. L. , Hollibaugh, J. T. , & Smith, S. V. (1997). Seasonally hypersaline estuaries in mediterranean‐climate regions. Estuarine, Coastal and Shelf Science, 45(6), 789–797. 10.1006/ecss.1997.0279

[jgrc25233-bib-0033] Lemagie, E. P. , Giddings, S. , MacCready, P. , Seaton, C. , & Wu, X. (2022). Data from: Measuring estuarine total exchange flow from discrete observations. UC San Diego Library Digital Collections. 10.6075/J0DJ5FS8 PMC9787582 36582261

[jgrc25233-bib-0034] Lemagie, E. P. , & Lerczak, J. A. (2014). A comparison of bulk estuarine turnover timescales to particle tracking timescales using a model of the Yaquina Bay estuary. Estuaries and Coasts, 38(5), 1797–1814. 10.1007/s12237-014-9915-1

[jgrc25233-bib-0035] Lerczak, J. , & Geyer, W. (2004). Modeling the lateral circulation in straight, stratified estuaries. Journal of Physical Oceanography, 34(6), 1410–1428. 10.1175/1520-0485(2004)034<1410:mtlcis>2.0.co;2

[jgrc25233-bib-0036] Lerczak, J. , Geyer, W. , & Chant, R. (2006). Mechanisms driving the time‐dependent salt flux in a partially stratified estuary. Journal of Physical Oceanography, 36(12), 2296–2311. 10.1175/jpo2959.1

[jgrc25233-bib-0037] Lorenz, M. , Klingbeil, K. , & Burchard, H. (2020). Numerical study of the exchange flow of the Persian Gulf using an extended total exchange flow analysis framework. Journal of Geophysical Research: Oceans, 125(2), e2019JC015527. 10.1029/2019JC015527

[jgrc25233-bib-0038] Lorenz, M. , Klingbeil, K. , MacCready, P. , & Burchard, H. (2019). Numerical issues of the total exchange flow (TEF) analysis framework for quantifying estuarine circulation. Ocean Science, 15(3), 601–614. 10.5194/os-15-601-2019

[jgrc25233-bib-0039] MacCready, P. (2011). Calculating estuarine exchange flow using isohaline coordinates. Journal of Physical Oceanography, 41(6), 1116–1124. 10.1175/2011JPO4517.1

[jgrc25233-bib-0040] MacCready, P. , Banas, N. S. , Hickey, B. M. , Dever, E. P. , & Liu, Y. (2009). A model study of tide‐ and wind‐induced mixing in the Columbia River estuary and plume. Continental Shelf Research, 29(1), 278–291. 10.1016/j.csr.2008.03.015

[jgrc25233-bib-0041] MacCready, P. , & Geyer, W. R. (2010). Advances in estuarine physics. Advances in estuarine physics, 2(1), 35–58. 10.1146/annurev-marine-120308-081015 21141657

[jgrc25233-bib-0042] MacCready, P. , Geyer, W. R. , & Burchard, H. (2018a). Estuarine exchange flow is related to mixing through the salinity variance budget. Journal of Physical Oceanography, 48(6), 1375–1384. 10.1175/JPO-D-17-0266.1

[jgrc25233-bib-0043] MacCready, P. , Geyer, W. R. , & Burchard, H. (2018b). Estuarine exchange flow is related to mixing through the salinity variance budget. Journal of Physical Oceanography, 48(6), 1375–1384. 10.1175/JPO-D-17-0266.1

[jgrc25233-bib-0044] MacCready, P. , & Giddings, S. N. (2016). The mechanical energy budget of a regional ocean model. Journal of Physical Oceanography, 46(9), 2719–2733. 10.1175/JPO-D-16-0086.1

[jgrc25233-bib-0045] MacCready, P. , McCabe, R. M. , Siedlecki, S. A. , Lorenz, M. , Giddings, S. N. , Bos, J. , et al. (2021). Estuarine circulation, mixing, and residence times in the Salish sea. Journal of Geophysical Research: Oceans, 126(2), e2020JC016738. 10.1029/2020JC016738

[jgrc25233-bib-0046] MacDonald, D. G. , & Horner‐Devine, A. R. (2008). Temporal and spatial variability of vertical salt flux in a highly stratified estuary. Journal of Geophysical Research, 113(C9), C09022. 10.1029/2007JC004620

[jgrc25233-bib-0047] Marshall, J. , Adcroft, A. , Hill, C. , Perelman, L. , & Heisey, C. (1997). A finite‐volume, incompressible Navier Stokes model for studies of the ocean on parallel computers. Journal of Geophysical Research, 102(C3), 5753–5766. 10.1029/96JC02775

[jgrc25233-bib-0048] Mazzini, P. L. F. , Barth, J. A. , Shearman, R. K. , & Erofeev, A. (2014). Buoyancy‐driven coastal currents off Oregon during fall and winter.

[jgrc25233-bib-0049] O'Callaghan, J. , Pattiaratchi, C. , & Hamilton, D. (2007). The response of circulation and salinity in a micro‐tidal estuary to sub‐tidal oscillations in coastal sea surface elevation. Continental Shelf Research, 27(14), 1947–1965. 10.1016/j.csr.2007.04.004

[jgrc25233-bib-0050] Reissmann, J. H. , Burchard, H. , Feistel, R. , Hagen, E. , Lass, H. U. , Mohrholz, V. , et al. (2009). Vertical mixing in the Baltic sea and consequences for eutrophication—A review. Progress in Oceanography, 82(1), 47–80. 10.1016/j.pocean.2007.10.004

[jgrc25233-bib-0051] Shchepetkin, A. F. , & McWilliams, J. C. (2005). The regional oceanic modeling system (ROMS): A split‐explicit, free‐surface, topography‐following‐coordinate oceanic model. Ocean Modelling, 9(4), 347–404. 10.1016/j.ocemod.2004.08.002

[jgrc25233-bib-0052] Simpson, J. H. , Crisp, D. J. , Hearn, C. , Swallow, J. C. , Currie, R. I. , Gill, A. E. , & Simpson, J. H. (1981). The shelf‐sea fronts: Implications of their existence and behaviour. Philosophical Transactions of the Royal Society of London ‐ Series A: Mathematical and Physical Sciences, 302(1472), 531–546. 10.1098/rsta.1981.0181

[jgrc25233-bib-0053] Sutherland, D. A. , MacCready, P. , Banas, N. S. , & Smedstad, L. F. (2011). A model study of the Salish sea estuarine circulation. Journal of Physical Oceanography, 41(6), 1125–1143. 10.1175/2011JPO4540.1

[jgrc25233-bib-0054] Thomson, R. E. , & Emery, W. J. (2014). Data analysis methods in physical oceanography (3edition ed.). Elsevier Science.

[jgrc25233-bib-0055] Valle‐Levinson, A. (2008). Density‐driven exchange flow in terms of the kelvin and ekman numbers. Journal of Geophysical Research, 113(C4), C04001. 10.1029/2007JC004144

[jgrc25233-bib-0056] Valle‐Levinson, A. , Reyes, C. , & Sanay, R. (2003). Effects of bathymetry, friction, and rotation on estuary–ocean exchange (Vol. 33, pp. 2375–2393).

[jgrc25233-bib-0057] Walin, G. (1977). A theoretical framework for the description of estuaries. Tellus, 29(2), 128–136. 10.3402/tellusa.v29i2.11337

[jgrc25233-bib-0058] Wang, T. , Geyer, W. R. , & MacCready, P. (2017). Total exchange flow, entrainment, and diffusive salt flux in estuaries. Journal of Physical Oceanography, 47(5), 1205–1220. 10.1175/JPO-D-16-0258.1

[jgrc25233-bib-0059] Warner, J. C. , Geyer, W. R. , & Arango, H. G. (2010). Using a composite grid approach in a complex coastal domain to estimate estuarine residence time. Computers & Geosciences, 36(7), 921–935. 10.1016/j.cageo.2009.11.008

[jgrc25233-bib-0060] Wei, Y. , Gille, S. T. , Mazloff, M. R. , Tamsitt, V. , Swart, S. , Chen, D. , & Newman, L. (2020). Optimizing mooring placement to constrain southern ocean air–sea fluxes. Journal of Atmospheric and Oceanic Technology, 37(8), 1365–1385. 10.1175/JTECH-D-19-0203.1

[jgrc25233-bib-0061] Westernik, J. J. , Luettich, R. A., Jr. , & Scheffner, N. (1993). Development of a tidal constituent database for the western north atlantic and gulf of Mexico. rep. 3, ADCIRC: An advanced three‐dimensional circulation model for shelves, coasts, and estuaries (No. DRP‐92‐6). Retrieved from https://apps.dtic.mil/sti/pdfs/ADA268685.pdf

[jgrc25233-bib-0062] Winant, C. D. (2004). Three‐dimensional wind‐driven flow in an elongated, rotating basin. Journal of Physical Oceanography, 34(2), 462–476. 10.1175/1520-0485(2004)034<0462:twfiae>2.0.co;2

[jgrc25233-bib-0063] Wu, X. , Feddersen, F. , & Giddings, S. N. (2021). Characteristics and dynamics of density fronts over the inner to midshelf under weak wind conditions. Journal of Physical Oceanography, 51(3), 789–808. 10.1175/JPO-D-20-0162.1

[jgrc25233-bib-0064] Wu, X. , Feddersen, F. , Giddings, S. N. , Kumar, N. , & Gopalakrishnan, G. (2020). Mechanisms of mid‐to outer‐shelf transport of shoreline‐released tracers. Journal of Physical Oceanography, 50(7), 1813–1837. 10.1175/JPO-D-19-0225.1

[jgrc25233-bib-0065] Zhang, Y. , & Baptista, A. M. (2008). SELFE: A semi‐implicit Eulerian–Lagrangian finite‐element model for cross‐scale ocean circulation. Ocean Modelling, 21(3), 71–96. 10.1016/j.ocemod.2007.11.005

